# Gene-Wide Analysis Detects Two New Susceptibility Genes for Alzheimer's Disease

**DOI:** 10.1371/journal.pone.0094661

**Published:** 2014-06-12

**Authors:** Valentina Escott-Price, Céline Bellenguez, Li-San Wang, Seung-Hoan Choi, Denise Harold, Lesley Jones, Peter Holmans, Amy Gerrish, Alexey Vedernikov, Alexander Richards, Anita L. DeStefano, Jean-Charles Lambert, Carla A. Ibrahim-Verbaas, Adam C. Naj, Rebecca Sims, Gyungah Jun, Joshua C. Bis, Gary W. Beecham, Benjamin Grenier-Boley, Giancarlo Russo, Tricia A. Thornton-Wells, Nicola Denning, Albert V. Smith, Vincent Chouraki, Charlene Thomas, M. Arfan Ikram, Diana Zelenika, Badri N. Vardarajan, Yoichiro Kamatani, Chiao-Feng Lin, Helena Schmidt, Brian Kunkle, Melanie L. Dunstan, Maria Vronskaya, the United Kingdom Brain Expression Consortium, Andrew D. Johnson, Agustin Ruiz, Marie-Thérèse Bihoreau, Christiane Reitz, Florence Pasquier, Paul Hollingworth, Olivier Hanon, Annette L. Fitzpatrick, Joseph D. Buxbaum, Dominique Campion, Paul K. Crane, Clinton Baldwin, Tim Becker, Vilmundur Gudnason, Carlos Cruchaga, David Craig, Najaf Amin, Claudine Berr, Oscar L. Lopez, Philip L. De Jager, Vincent Deramecourt, Janet A. Johnston, Denis Evans, Simon Lovestone, Luc Letenneur, Isabel Hernández, David C. Rubinsztein, Gudny Eiriksdottir, Kristel Sleegers, Alison M. Goate, Nathalie Fiévet, Matthew J. Huentelman, Michael Gill, Kristelle Brown, M. Ilyas Kamboh, Lina Keller, Pascale Barberger-Gateau, Bernadette McGuinness, Eric B. Larson, Amanda J. Myers, Carole Dufouil, Stephen Todd, David Wallon, Seth Love, Ekaterina Rogaeva, John Gallacher, Peter St George-Hyslop, Jordi Clarimon, Alberto Lleo, Anthony Bayer, Debby W. Tsuang, Lei Yu, Magda Tsolaki, Paola Bossù, Gianfranco Spalletta, Petra Proitsi, John Collinge, Sandro Sorbi, Florentino Sanchez Garcia, Nick C. Fox, John Hardy, Maria Candida Deniz Naranjo, Paolo Bosco, Robert Clarke, Carol Brayne, Daniela Galimberti, Elio Scarpini, Ubaldo Bonuccelli, Michelangelo Mancuso, Gabriele Siciliano, Susanne Moebus, Patrizia Mecocci, Maria Del Zompo, Wolfgang Maier, Harald Hampel, Alberto Pilotto, Ana Frank-García, Francesco Panza, Vincenzo Solfrizzi, Paolo Caffarra, Benedetta Nacmias, William Perry, Manuel Mayhaus, Lars Lannfelt, Hakon Hakonarson, Sabrina Pichler, Minerva M. Carrasquillo, Martin Ingelsson, Duane Beekly, Victoria Alvarez, Fanggeng Zou, Otto Valladares, Steven G. Younkin, Eliecer Coto, Kara L. Hamilton-Nelson, Wei Gu, Cristina Razquin, Pau Pastor, Ignacio Mateo, Michael J. Owen, Kelley M. Faber, Palmi V. Jonsson, Onofre Combarros, Michael C. O'Donovan, Laura B. Cantwell, Hilkka Soininen, Deborah Blacker, Simon Mead, Thomas H. Mosley, David A. Bennett, Tamara B. Harris, Laura Fratiglioni, Clive Holmes, Renee F. A. G. de Bruijn, Peter Passmore, Thomas J. Montine, Karolien Bettens, Jerome I. Rotter, Alexis Brice, Kevin Morgan, Tatiana M. Foroud, Walter A. Kukull, Didier Hannequin, John F. Powell, Michael A. Nalls, Karen Ritchie, Kathryn L. Lunetta, John S. K. Kauwe, Eric Boerwinkle, Matthias Riemenschneider, Mercè Boada, Mikko Hiltunen, Eden R. Martin, Reinhold Schmidt, Dan Rujescu, Jean-François Dartigues, Richard Mayeux, Christophe Tzourio, Albert Hofman, Markus M. Nöthen, Caroline Graff, Bruce M. Psaty, Jonathan L. Haines, Mark Lathrop, Margaret A. Pericak-Vance, Lenore J. Launer, Christine Van Broeckhoven, Lindsay A. Farrer, Cornelia M. van Duijn, Alfredo Ramirez, Sudha Seshadri, Gerard D. Schellenberg, Philippe Amouyel, Julie Williams

**Affiliations:** 1 Institute of Psychological Medicine and Clinical Neurosciences, MRC Centre for Neuropsychiatric Genetics & Genomics, Cardiff University, Cardiff, United Kingdom; 2 Inserm U744, Lille, France; 3 Université Lille 2, Lille, France; 4 Institut Pasteur de Lille, Lille, France; 5 Department of Pathology and Laboratory Medicine, University of Pennsylvania Perelman School of Medicine, Philadelphia, Pennsylvania, United States of America; 6 Department of Biostatistics, Boston University School of Public Health, Boston, Massachusetts, United States of America; 7 Department of Epidemiology and Neurology, Erasmus MC University Medical Center, Rotterdam, the Netherlands; 8 Department of Biostatistics and Epidemiology and Center for Clinical Epidemiology and Biostatistics, Perelman School of Medicine, University of Pennsylvania, Philadelphia, Pennsylvania, United States of America; 9 Department of Medicine (Biomedical Genetics), Boston University School of Medicine, Boston, Massachusetts, United States of America; 10 Department of Ophthalmology, Boston University School of Medicine, Boston, Massachusetts, United States of America; 11 Cardiovascular Health Research Unit, Department of Medicine, University of Washington, Seattle, Washington, United States of America; 12 The John P. Hussman Institute for Human Genomics, University of Miami, Miami, Florida, United States of America; 13 Dr. John T. Macdonald Foundation Department of Human Genetics, University of Miami, Miami, Florida, United States of America; 14 Functional Genomics Center Zurich, ETH/University of Zurich, Zurich, Switzerland; 15 Department of Molecular Physiology and Biophysics, Vanderbilt University, Nashville, Tennessee, United States of America; 16 University of Iceland, Faculty of Medicine, Reykjavik, Iceland; 17 Icelandic Heart Association, Kopavogur, Iceland; 18 Department of Neurology, Boston University School of Medicine, Boston, Massachusetts, United States of America; 19 Departments of Epidemiology, Neurology and Radiology, Erasmus MC University Medical Center, Rotterdam, the Netherlands; 20 Netherlands Consortium for Healthy Aging, Leiden, The Netherlands; 21 Centre National de Genotypage, Institut Genomique, Commissariat à l'énergie Atomique, Evry, France; 22 Fondation Jean Dausset- CEPH, Paris, France; 23 Institute for Molecular Biology and Biochemistry, Medical University of Graz, Graz, Austria; 24 Reta Lila Weston Research Laboratories, Department of Molecular Neuroscience, UCL Institute of Neurology, London, United Kingdom; 25 NHLBI Cardiovascular Epidemiology and Human Genomics Branch, The Framingham Heart Study, Framingham, Massachusetts, United States of America; 26 Memory Clinic of Fundació ACE. Institut Català de Neurociències Aplicades, Barcelona, Spain; 27 Taub Institute on Alzheimer's Disease and the Aging Brain, Department of Neurology, Columbia University New York, New York, United States of America; 28 Gertrude H. Sergievsky Center, Department of Neurology, Columbia University, New York, New York, United States of America; 29 CNR-MAJ, Centre Hospitalier Régional Universitaire de Lille, Lille, France; 30 University Paris Descartes, Sorbonne Paris V, Broca Hospital, Geriatrics department, Paris, France; 31 Departments of Epidemiology and Global Health, University of Washington, Seattle, Washington, United States of America; 32 Department of Neuroscience, Mount Sinai School of Medicine, New York, New York, United States of America; 33 Department of Psychiatry, Mount Sinai School of Medicine, New York, New York, United States of America; 34 Departments of Genetics and Genomic Sciences, Mount Sinai School of Medicine, New York, New York, United States of America; 35 CNR-MAJ, Inserm U1079, Rouen University Hospital, 76031 France, Rouen, France; 36 Department of Medicine, University of Washington, Seattle, Washington, United States of America; 37 German Center for Neurodegenerative Diseases (DZNE), Bonn, and Institute for Medical Biometry, Informatics and Epidemiology, University of Bonn, Bonn, Germany; 38 Department of Psychiatry and Hope Center Program on Protein Aggregation and Neurodegeneration, Washington University School of Medicine, St. Louis, Missouri, United States of America; 39 Ageing Group, Centre for Public Health, School of Medicine, Dentistry and Biomedical Sciences, Queen's University Belfast, Belfast, United Kingdom; 40 Department of Epidemiology, Erasmus MC University Medical Center, Rotterdam, the Netherlands; 41 INSERM U1061, Faculty of Medicine, Hôpital La Colombière, Montpellier, France; 42 Departments of Neurology, University of Pittsburgh School of Medicine, Pittsburgh, Pennsylvania, United States of America; 43 Program in Translational NeuroPsychiatric Genomics, Institute for the Neurosciences, Department of Neurology & Psychiatry, Brigham and Women's Hospital and Harvard Medical School, Boston, Massachusetts, United States of America; 44 Program in Medical and Population Genetics, Broad Institute, Boston, Massachusetts, United States of America; 45 Rush Institute for Healthy Aging, Department of Internal Medicine, Rush University Medical Center, Chicago, Illinois, United States of America; 46 King's College London, Institute of Psychiatry, Department of Neuroscience, De Crespigny Park, Denmark Hill, London, United Kingom; 47 Inserm U897, Victor Segalen University, F-33076, Bordeaux, France; 48 Cambridge Institute for Medical Research, University of Cambridge, Cambridge, United Kingdom; 49 Neurodegenerative Brain Diseases Group, Department of Molecular Genetics, VIB, Antwerp, Belgium; 50 Laboratory of Neurogenetics, Institute Born-Bunge, University of Antwerp, Antwerp, Belgium; 51 Neurogenomics Division, Translational Genomics Research Institute, Phoenix, Arizona, United States of America; 52 Discipline of Psychiatry, Trinity College, Dublin, Ireland; 53 Institute of Genetics, Queen's Medical Centre, University of Nottingham, Nottingham, United Kingdom; 54 Department of Human Genetics, University of Pittsburgh, Pittsburgh, Pennsylvania, United States of America; 55 Alzheimer's Disease Research Center, University of Pittsburgh, Pittsburgh, Pennsylvania, United States of America; 56 Aging Reasearch Center, Department of Neurobiology, Care Sciences and Society, Karolinska Institutet and Stockholm University, Stockholm, Sweden; 57 Group Health Research Institute, Group Health, Seattle, Washington, United States of America; 58 Department of Psychiatry and Behavioral Sciences, Miller School of Medicine, University of Miami, Miami, Florida, United States of America; 59 University of Bristol Institute of Clinical Neurosciences, School of Clinical Sciences, Frenchay Hospital, Bristol, United Kingdom; 60 Tanz Centre for Research in Neurodegenerative Disease, University of Toronto, Toronto, Ontario, Canada; 61 Institute of Primary Care and Public Health, Cardiff University, Neuadd Meirionnydd, University Hospital of Wales, Heath Park, Cardiff, United Kingdom; 62 Cambridge Institute for Medical Research and Department of Clinical Neurosciences, University of Cambridge, Cambridge, United Kingdom; 63 Neurology Department. IIB Sant Pau. Sant Pau Hospital. Universitat Autònoma de Barcelona, Barcelona, Spain; 64 Center for Networker Biomedical Research in Neurodegenerative Diseases (CIBERNED), Barcelona, Spain; 65 Department of Psychiatry and Behavioral Sciences, University of Washington, Seattle, Washington, United States of America; 66 Department of Neurological Sciences, Rush University Medical Center, Chicago, Illinois, United States of America; 67 3rd Department of Neurology, Aristotle University of Thessaloniki, Thessaloniki, Greece; 68 Clinical and Behavioral Neurology, Fondazione Santa Lucia, Roma, Italy; 69 MRC Prion Unit, Department of Neurodegenerative Disease, UCL Institute of Neurology, London, United Kingdom; 70 NEUROFARBA Department of Neuroscience, Psychology, Drug Research and Child Health, University of Florence, Florence, Italy; 71 Centro di Ricerca, Trasferimento e Alta Formazione DENOTHE, University of Florence, Florence, Italy; 72 Department of Immunology, Hospital Universitario Dr. Negrin, Las Palmas de Gran Canaria, Spain; 73 Dementia Research Center, Department of Neurodegenerative Disease, UCL Institute of Neurology, London, United Kingdom; 74 Department of Molecular Neuroscience and Reta Lilla Weston Laboratories, Institute of Neurology, London, United Kingdom; 75 IRCCS Associazione Oasi Maria SS, Troina, Italy; 76 Oxford Healthy Aging Project (OHAP), Clinical Trial Service Unit, University of Oxford, Oxford, United Kingdom; 77 Cognitive Function and Ageing Study (CFAS), Institute of Public Health, University of Cambridge, Cambridge, United Kingdom; 78 University of Milan, Fondazione Cà Granda, IRCCS Ospedale Policlinico, Milan, Italy; 79 Neurological Clinic, University of Pisa, Pisa, Italy; 80 Urban Epidemiology, Institute for Medical Informatics, Biometry and Epidemiology, University Hospital Essen, University Duisburg-Essen, Essen, Germany; 81 Section of Gerontology and Geriatrics, Department of Clinical and Experimental Medicine, University of Perugia, Perugia, Italy; 82 Section of Neuroscience and Clinical Pharmacology, Department of Biomedical Sciences, University of Cagliari, Cagliari, Italy; 83 Department of Psychiatry and Psychotherapy, University of Bonn, Germany and German Center for Neurodegenerative Diseases (DZNE, Bonn), Bonn, Germany; 84 Department of Psychiatry, University of Frankfurt am Main, Frankfurt am Main, Germany (H.H.); 85 Department of Psychiatry, Ludwig-Maximilians University, Munich, Germany; 86 Gerontology and Geriatrics Research Laboratory, I.R.C.C.S. Casa Sollievo della Sofferenza, San Giovanni Rotondo (FG), Italy; 87 Centro de Biología Molecular Severo Ochoa (CSIC-UAM); Madrid, Spain; 88 Centro de Investigación Biomédica en Red sobre Enfermedades Neurodegenerativas (CIBERNED), Madrid, Spain; 89 Instituto de Investigación Sanitaria “Hospital la Paz” (IdIPaz), Madrid, Spain; 90 Department of Geriatrics,Center for Aging Brain,University of Bari, Bari, Italy; 91 Department of Neuroscience-University of Parma, Parma, Italy; 92 Center for Cognitive Disorders AUSL, Parma, Italy; 93 Department Of Psychiatry, University Hospital, Saarland, Germany; 94 Department of Public Health/Geriatrics, Uppsala University, Uppsala, Sweden; 95 Center for Applied Genomics, Children's Hospital of Philadelphia, Philadelphia, Pennsylvania, United States of America; 96 Department of Neuroscience, Mayo Clinic, Jacksonville, Florida, United States of America; 97 National Alzheimer's Coordinating Center, University of Washington, Seattle, Washington, United States of America; 98 Genetica molecular-Huca-Oviedo, Oviedo, Spain; 99 Department of Psychiatry, University Hospital, Saarland, Germany; 100 Neurogenetics Laboratory, Division of Neurosciences, Center for Applied Medical Research, University of Navarra School of Medicine, Pamplona, Spain; 101 CIBERNED, Centro de Investigación Biomédica en Red de Enfermedades Neurodegenerativas, Instituto de Salud Carlos III, Madrid, Spain; 102 Neurology Service and CIBERNED, "Marqués de Valdecilla" University Hospital (University of Cantabria and IFIMAV), Santander, Spain; 103 Department of Medical and Molecular Genetics, Indiana University, Indianapolis, Indiana, United States of America; 104 Landspitali University Hospital, Reykjavik, Iceland; 105 Institute of Clinical Medicine - Neurology, University of Eastern Finland, Kuopio, Finland; 106 Department of Neurology, Kuopio University Hospital, Kuopio, Finland; 107 Department of Epidemiology, Harvard School of Public Health, Boston, Massachusetts, United States of America; 108 Department of Psychiatry, Massachusetts General Hospital/Harvard Medical School, Boston, Massachusetts, United States of America; 109 Department of Medicine (Geriatrics), University of Mississippi Medical Center, Jackson, Mississippi, United States of America; 110 Rush Alzheimer's Disease Center, Rush University Medical Center, Chicago, Illinois, United States of America; 111 Laboratory of Epidemiology, Demography, and Biometry, National Institute of Health, Bethesda, Maryland, United States of America; 112 Aging Research Center, Department Neurobiology, Care Sciences and Society, Karolinska Institutet and Stockholm University, Stockholm, Sweden; 113 Department Geriatric Medicine, Genetics Unit, Karolinska University Hospital Huddinge, Stockholm, Sweden; 114 Division of Clinical Neurosciences, School of Medicine, University of Southampton, Southampton, United Kingdom; 115 Departments of Neurology and Epidemiology, Erasmus MC University Medical Center, Rotterdam, the Netherlands; 116 Department of Pathology, University of Washington, Seattle, Washington, United States of America; 117 Institute for Translational Genomics and Population Sciences, Los Angeles Biomedical Research Institute at Harbor-UCLA Medical Center, Torrance, California, United States of America; 118 INSERM UMR_S975-CNRS UMR 7225, Université Pierre et Marie Curie, Centre de recherche de l'Institut du Cerveau et de la Moëlle épinière-CRICM, Hôpital de la Salpêtrière, Paris France; 119 AP-HP, Hôpital de la Pitié-Salpêtrière, Paris, France; 120 Department of Epidemiology, University of Washington, Seattle, Washington, United States of America; 121 Laboratory of Neurogenetics, Intramural Research Program, National Institute on Aging, Bethesda, Maryland, United States of America; 122 Imperial College, London, United Kingdom; 123 Department of Biology, Brigham Young University, Provo, Utah, United States of America; 124 Human Genome Sequencing Center, Baylor College of Medicine, Houston, Texas, United States of America; 125 Human Genetics Center and Div. of Epidemiology, University of Texas Health Sciences Center at Houston, Houston, Texas, United States of America; 126 Hospital Universitari Vall d'Hebron - Institut de Recerca, Universitat Autònoma de Barcelona. (VHIR-UAB), Barcelona, Spain; 127 Department of Neurology, Medical University Graz, Graz, Austria; 128 Centre de Mémoire de Ressources et de Recherche de Bordeaux, CHU de Bordeaux, Bordeaux, France; 129 Inserm U708, Victor Segalen University, Bordeaux, France; 130 Institute of Human Genetics, Department of Genomics, Life and Brain Center, University of Bonn, and German Center for Neurodegenerative Diseases (DZNE, Bonn), Bonn, Germany; 131 Karolinska Institutet, Department of Neurobiology, Care Sciences and Society, KIADRC, Stockholm, Sweden; 132 Group Health Research Institute, Group Health Cooperative, Seattle, Washington, United States of America; 133 Vanderbilt Center for Human Genetics Research, Vanderbilt University, Nashville, Tennessee, United States of America; 134 Department of Epidemiology & Biostatistics, Case Western Reserve University, Cleveland, Ohio, United States of America; 135 McGill University and Génome Québec Innovation Centre, Montreal, Canada; 136 Department of Epidemiology, Boston University School of Public Health, Boston, Massachusetts, United States of America; 137 Department of Neurology, Boston University School of Medicine, Boston, Massachusetts, United States of America; 138 Center for Medical Systems Biology, Leiden, The Netherlands; 139 Department of Psychiatry and Psychotherapy and Institute of Human Genetics, University of Bonn, Bonn, Germany; 140 The Framingham Heart Study, Framingham, Massachusetts, United States of America; 141 Centre Hospitalier Régional Universitaire de Lille, Lille, France; Kunming Institute of Zoology, Chinese Academy of Sciences, China

## Abstract

**Background:**

Alzheimer's disease is a common debilitating dementia with known heritability, for which 20 late onset susceptibility loci have been identified, but more remain to be discovered. This study sought to identify new susceptibility genes, using an alternative gene-wide analytical approach which tests for patterns of association within genes, in the powerful genome-wide association dataset of the International Genomics of Alzheimer's Project Consortium, comprising over 7 m genotypes from 25,580 Alzheimer's cases and 48,466 controls.

**Principal Findings:**

In addition to earlier reported genes, we detected genome-wide significant loci on chromosomes 8 (*TP53INP1*, p = 1.4×10^−6^) and 14 (*IGHV1-67* p = 7.9×10^−8^) which indexed novel susceptibility loci.

**Significance:**

The additional genes identified in this study, have an array of functions previously implicated in Alzheimer's disease, including aspects of energy metabolism, protein degradation and the immune system and add further weight to these pathways as potential therapeutic targets in Alzheimer's disease.

## Introduction

The prevalence of Alzheimer's disease (AD) is increasing as more people live into old age. Hope for finding preventative and clinical therapies lies in the ability to gain a better understanding of the underlying biology of the disease, and genetics will provide a valuable starting point for advancement. Rare monogenic forms of AD, the majority of which are attributable to mutations in one of three genes, *APP*, *PSEN1* and *PSEN2*, exist, but common, late-onset AD is genetically complex with heritability estimated to be between 56–79%[1], [2]. Along with the APOE polymorphism[3], 20 common susceptibility loci have been identified associated with AD[4]–[9]. (This figure does not include *CD33* as it did not show genome-wide significance in the original report[9].) Recently, a moderately rare variant in *TREM2* has also shown evidence for association[10]. However, new variants remain to be found. This study sought to identify new susceptibility genes, using an alternative gene-wide analytical approach, which focuses on the pattern of association within gene regions.

Genome-wide association (GWA) studies to date have focused on single nucleotide polymorphisms (SNPs) as the unit of analysis. Single locus tests are the simplest to generate and to interpret, but have limitations. For example, if susceptibility is conferred by multiple variants within a locus[11], [12], this gives rise to complex patterns of association that might not be reflected by association to the same SNPs in different samples, despite apparently reasonably powered tests[13], [14]. In addition, rare risk-increasing variants may not be tagged by single SNPs, as is e.g. the case for *CLU* in which significant enrichment of rare variants in patients was observed independent of the single locus GWA signal[15]. It is therefore likely that the power to detect association might be enhanced by exploiting information from multiple signals within genes encompassed by gene-wide statistical approaches[12]. Disease risk may reflect the co-action of several loci but the number of loci involved at the individual or the population levels are unknown, as is the spectrum of allele frequencies and effect sizes[16]. The observations of multiple genome-wide significant or suggestive linkage signals for disorders, that do not readily replicate between studies but which are not randomly distributed across the genome[17], [18] is compatible with the existence of multiple risk alleles of moderate effect that would implicate a locus in disease risk, when analysed together. Thus the first aim of this study is to test for gene-wide association with AD, using a powerful mega-meta analysis of genome-wide datasets as part of the International Genomics of Alzheimer's Project (IGAP) Consortium comprising four AD genetic consortia (see the full list of consortia members in Materials S1): Genetic and Environmental Risk in Alzheimer's Disease (GERAD), European Alzheimer's Disease Initiative (EADI), Cohorts for Heart and Aging in Genomic Epidemiology (CHARGE) and Alzheimer's Disease Genetics Consortium (ADGC) (see full IGAP datasets description in Materials S2). A two stage study was undertaken. In Stage 1 the combined sample included 17,008 AD cases and 37,154 controls. In Stage 2 loci with p-values (combined over all SNPs at the locus) less than 10^−4^ were selected for replication for 8,572 AD cases and 11,312 controls of European ancestry. We observed evidence for gene-wide association at loci which implicate genes which already show genome-wide significant association from single SNP analysis (*CR1, BIN1, HLA-DRB5/HLA-DRB1, CD2AP, EPHA1, PTK2B, CLU, MS4A6A, PICALM, SORL1, SLC24A4, ABCA7, APOE*), three new genes in the vicinity of lately reported single SNP hits[9] (*ZNF3*, *NDUFS3, MTCH2*) and two novel loci (*TP53INP1*, combined p = 1.4×10^−6^ and *IGHV1-67* combined p = 7.9×10^−8^).

## Results

Initially, we tested for excess genetic signal revealed by the Stage 1 IGAP SNP GWAS study. We observed more SNPs at all significance intervals, and more genes at multiple significance thresholds, than expected by chance (Table S1). This is unlikely to be due to uncorrected stratification, since each of the individual GWAS samples in the IGAP Stage 1 analysis was corrected for ethnic variation. Thus it is likely that the sample contains novel genetic signals, in addition to those detected by the primary analysis[9], [19].

Next, we looked at overrepresentation of significant genes in the Stage 1 data. Table 1 gives the observed and expected numbers of significant genes at significance levels 10^−4^, 10^−5^, 10^−6^ when all genes are counted in the analyses and when the known genes (Table S1) and genes within 500kb of them are excluded, the observed numbers of genes are much larger than expected at all significance levels (all p≤0.001). Thus there are more loci associated with AD to find.

**Table 1:** Overrepresentation of replication of significant genes/loci available at Stage 2, excluding all loci of 0.5 Mb around genes previously reported[4]–[8] and Stage 1 IGAP genes[9], [19] containing genome-wide significant SNPs.

	GENES		LOCI		
Stage 1 significance level	Significant at Stage 1	Replicated (p≤0.05) at Stage 2	Significant at Stage 1	Replicated (p≤0.05) at Stage 2	Over-representation p-value
p≤10^−4^	27	9 (33%)	9	3 (33%)	0.109
p≤10^−3^	74	17 (23%)	36	8 (22%)	0.125
p≤0.01	229	49 (21%)	102	26 (25%)	0.0001
p≤0.05	390	77 (20%)	171	33 (19%)	0.007
Total (p≤1)	887	124 (14%)	444	60 (13.5%)	4.6×10^−12^

Over-representation p-values were calculated with chi-square/Fisher's exact tests counting the genes within 0.5 Mb as one locus.

Furthermore, the number of independent nominally significant loci at Stage 2 (N = 60, (13.5%)) was significantly greater than expected by chance (p = 4.6×10^−12^). The percentage of replicated loci increased with the decrease of the gene-wise significance threshold at Stage 1 (see Table 2 for details).

**Table 2:** Overrepresentation of significant loci, excluding regions of 0.5[4]–[8] and Stage 1 IGAP genes[9], [19] containing genome-wide significant SNPs.

	Numbers of loci (genes)		
	p≤10^−4^	p≤10^−5^	p≤10^−6^
Observed	9(27)	4(8)	2(2)
Expected	2.5	0.25	0.025
p-value	0.001	0.00013	0.0003

The observed number of genes is calculated by combining significant loci within 0.5 Mb into one signal. The APOE region is excluded (CHR19; 44,411,940–46,411,945bp). The total number of genes after exclusions is 24,849.

**Figure 1:**
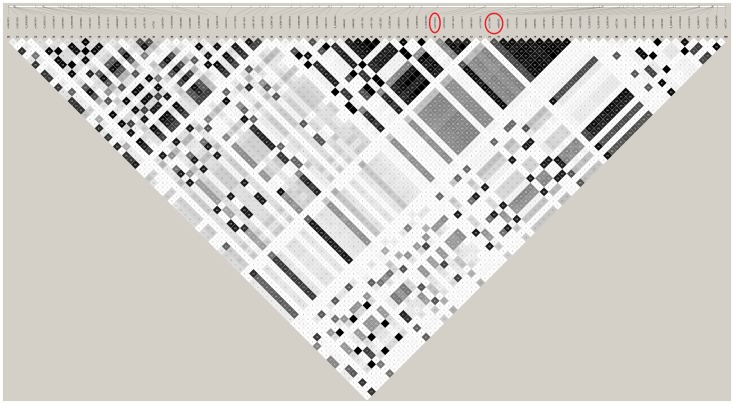
Linkage disequilibrium structure of *TP53INP1* gene. The SNPs which are significant at 10^−4^ level are circled in red.

Combining the gene-wide p-values in both stages 1 and 2, using Fisher's method revealed two new gene-based genome-wide significant (p<2.5×10^−6^) loci *TP53INP1* and *IGHV1-67*. The *TP53INP1* gene is located on chromosome 8∶95,938,200–95,961,615 and its combined gene-based p-value = 1.4×10^−6^ (Table 3). Table S3 provides details for each SNP contributing to the gene-based result. Out of 45 SNPs in the gene, three SNPs (rs4735333, rs1713669, rs896855) have p-value≤10^−4^. Figure 1 shows the LD plot of this gene and suggests that there are at least two partially independent signals in the *TP53INP1* gene (r^2^ between the pairs of most significant SNPs rs4735333-rs1713669 and rs1713669- rs896855 are 0.65 and 0.6 respectively).

**Table 3:** New genome-wide significant genes associated with AD.

Gene Name	Chr	Position	Stage 1 gene-wide p-value	Stage 2 gene-wide p-value	N of SNPs per gene	Combined gene-wide p-value	Combined best SNP p-value	Biological function
*TP53INP1*	8	95,938,200–95,961,615	1.7×10^−2^	4.5×10^−3^	45	1.4×10^−6^	1.5×10^−7^	Regulation of autophagy, cell cycle arrest
*IGHV1-67*	14	107,136,620–107,137,059	2.3×10^−4^	3.2×10^−5^	2	7.9×10^−8^	3.9×10^−5^	Immunoglobulin heavy chain region: adaptive immunity
New genes in the vicinity of recently reported single SNP genome-wide significant hits[9], [19]:								
*ZNF3*	7	99,661,653–99,679,371	2.7×10^−2^	1.8×10^−6^	27	8.6×10^−7^	3.1×10^−7^	Transcription factor, leucocyte activation
*NDUFS3*	11	47,600,632–47,606,114	1.2×10^−6^	2.2×10^−2^	5	4.8×10^−7^	2.9×10^−6^	Mitochondrial electron transport, NADH to ubiquinone
*MTCH2*	11	47,638,858–47,664,206	1.7×10^−5^	8.7×10^−3^	34	2.5×10^−6^	7.2×10^−8^	Mitochondrial inner membrane

Gene-wide p-values are shown for those genes with p<2.5×10^−6^ for which the best single-SNP p-value in that gene is greater than 5×10^−8^ in the combined Stage 1 and Stage 2 sample. Previously reported genes[4]–[8] ± 0.5 Mb around them are excluded.

Gene-wide p-values in the combined Stage 1 and Stage 2 sample obtained by combining the p-values from the Stage 1 with those from the Stage 2 using Fisher's method.

**Figure 2:**
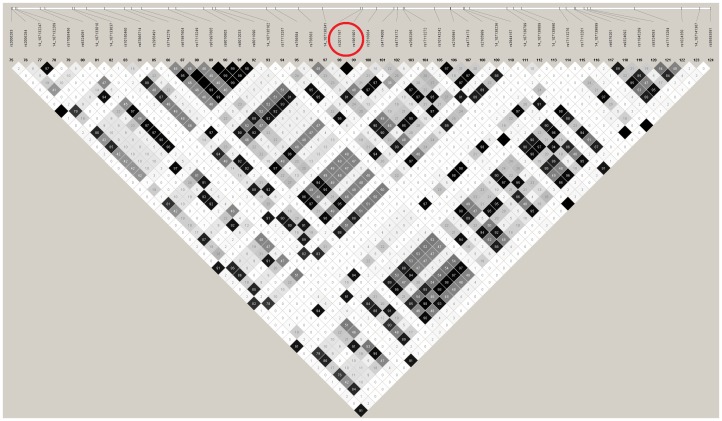
Linkage disequilibrium structure of *IGHV1-67* gene ±5 kb. The SNPs which are significant at 10^−4^ level are circled in red.

The *IGHV1-67* gene on chromosome 14∶107,136,620–107,137,059 has combined p-value = 7.9×10^−8^ (Tables 3). This gene is covered by two SNPs (rs2011167, rs1961901), both are significant at 10^−4^ level. LD plot in Figure 2 and Table S4 indicate that the two most significant SNPs in *IGHV1-67* gene represent almost the same signal (r^2^ = 0.92, calculated with SNAP software[20], 1000 genomes Pilot 1 dataset, CEU population panel, (http://www.broadinstitute.org/mpg/snap)).

To look at the gene expression patterns in these novel genes, we used the Webster-Myers expression dataset[21], available at http://labs.med.miami.edu/myers/LFuN/data%20ajhg.html. Comparing 137 AD vs 176 controls with temporal or frontal cortex expression values by t-test, t showed significantly higher TP53INP1 expression in cases compared to controls (p = 0.0128). Further examination in the BRAINEAC database[22] (www.braineac.org) from the UK Brain Expression Consortium showed *TP53INP1* to have a best cis-eQTL p-value of 6.8×10^−6^ (for rs4582532 SNP, which is about 7.6 kb upstream of the gene). The three SNPs with association p≤10^−4^ mentioned above (rs4735333, rs1713669, rs896855) had significant cis-eQTL p-values of 8.2×10^−6^, 7.8×10^−5^ and 1.1×10^−5^ respectively in BRAINEAC brain expression data. The r^2^ between the cis-eQTL and the three associated SNPs were 0.80, 0.65, and 0.81, respectively). Further analysis of additional independent brain expression and methylation datasets (see Methods S1) indicated significant *cis* eQTLs and meQTLs for *TP53INP1* (Tables S10 and S11). The probe for the meQTL is in a CpG island region that corresponds well with ENCODE DNAse/ChIP-seq/Histone marks and is located upstream (∼1.5 kb) of the *TP53INP1* transcription start site. In combination these results suggest a possible epigenetic mechanism whereby the associated variants in the region influence *TP53INP1* expression in several brain regions. These expression data provide further evidence supporting the functional relevance of *TP53INP1* to AD susceptibility. The *IGHV1-67* gene was not found in those databases.

In addition we detected two genome-wide significant loci 1) *ZNF3* (chr7: 99,661,653–99,679,371; p = 8.6×10^−7^) and 2) two closely located genes on chromosome 11 *MTCH2* (47,638,858–47,664,206, combined p = 2.5×10^−6^) and *NDUFS3* (47,600,632–47,606,114, combined p = 4.8×10^−7^) (Table 4). None of these genes harbour genome-wide significant SNPs in the SNP GWAS analysis on its own (see Tables S5-S7). Figures S1-S3 show LD plots of these additional genes.

**Table 4:** New genome-wide significant genes associated with AD in the vicinity of recently reported single SNP genome-wide significant hits[9], [19].

Gene Name	Chr	Position	Stage 1 gene-wide p-value	Stage 2 gene-wide p-value	N of SNPs per gene	Combined gene-wide p-value	Combined best SNP p-value	Biological function
*ZNF3*	7	99,661,653–99,679,371	2.7×10^−2^	1.8×10^−6^	27	8.6×10^−7^	3.1×10^−7^	Transcription factor, leucocyte activation
*NDUFS3*	11	47,600,632–47,606,114	1.2×10^−6^	2.2×10^−2^	5	4.8×10^−7^	2.9×10^−6^	Mitochondrial electron transport, NADH to ubiquinone
*MTCH2*	11	47,638,858–47,664,206	1.7×10^−5^	8.7×10^−3^	34	2.5×10^−6^	7.2×10^−8^	Mitochondrial inner membrane

Gene-wide p-values are shown for those genes with p<2.5×10^−6^ for which the best single-SNP p-value in that gene is greater than 5×10^−8^ in the combined Stage 1 and Stage 2 sample. Previously reported genes[4]–[8] ± 0.5 Mb around them are excluded.

Gene-wide p-values in the combined Stage 1 and Stage 2 sample obtained by combining the p-values from the Stage 1 with those from the Stage 2 using Fisher's method. The LD between rs1476679 (chr7∶100,004,446) reported by IGAP [9] and the best SNP in ZNF3 is r^2^ = 0.16. The LD between rs10838725 (chr11: 47,557,871) reported by IGAP [9] and the best SNPs in the region on chr 11 in the table are r^2^ = 0.3 and 0.88 for *NDUFS3* and *MTCH2* respectively.

*ZNF3* and *NDUFS3*, *MTCH2* genes on chromosomes 7 and 11, respectively, lie close to rs1476679 (chr7∶100,004,446; *ZCWPW1*) and rs1083872 (chr11∶47,557,871; *CELF1*) SNPs, which are shown to be genome-wide significant in the IGAP study, when combining Stage 1 and Stage 2 data. Figures S1-S3 show LD structure of these genes in relation to the IGAP singe genome-wide significant hits. (Note that the *NDUFS3* gene on chromosome 11 was gene-based genome-wide significant already at Stage 1.) Although none of these SNPs actually lie within the genes mentioned above, it is possible that they may account for the gene-based signals through linkage disequilibrium. In order to test whether the gene-based signals are independent of these strongly-associated SNPs, we performed single-SNP association for each SNP annotated to these genes by regression, adjusting for the significant SNPs mentioned above, along with the other study covariates. The resulting p-values were combined into gene-based tests, as described previously. Under this conditional analysis *ZNF3* gene does not show significant association, however *NDUFS3* still shows a trend towards significance (p = 0.081) (see Table S8 for details). Furthermore, five genes in chr11∶47,593,749–47,615,961 (*KBTBD4, NDUFS3, LOC100287127, FAM180B, C1QTNF4*) all have p<0.05 with gene-based analysis ±10 kb, when conditioning by the genome-wide significant hit rs10838725 in this region. This may partially be explained by the SNP rs10838731 (p = 1.2×10^−3^ after conditioning by rs10838725) which is shared by all latter five genes.

Gene-based analysis with ±10 kb around genes did not reveal additional genome-wide significant loci in the Stage 1 data set. Moreover, the significance of the genes identified above did not improve in general, indicating that adding 10 kb flanking regions to genes introduces more noise to the gene-based signal. The combined Stage 1 and Stage 2 gene-based analysis provided further evidence for significant signals in the loci on chr 11 with 8 genes (*SPI1, SLC39A13*, *LOC100287086*, *PTPMT1*, *KBTBD4, NDUFS3, LOC100287127, FAM180B*) and on chr 7 with 6 genes (*LOC100128334*, *MCM7*, *PILRB*, *PILRA*, *LOC100289298*, *C7orf51*), all reaching genome-wide significance. This is likely to be due to the fact that including genes' flanking regions captures a greater number of the same SNPs or SNPs in high LD showing significant association.

**Figure 3:**
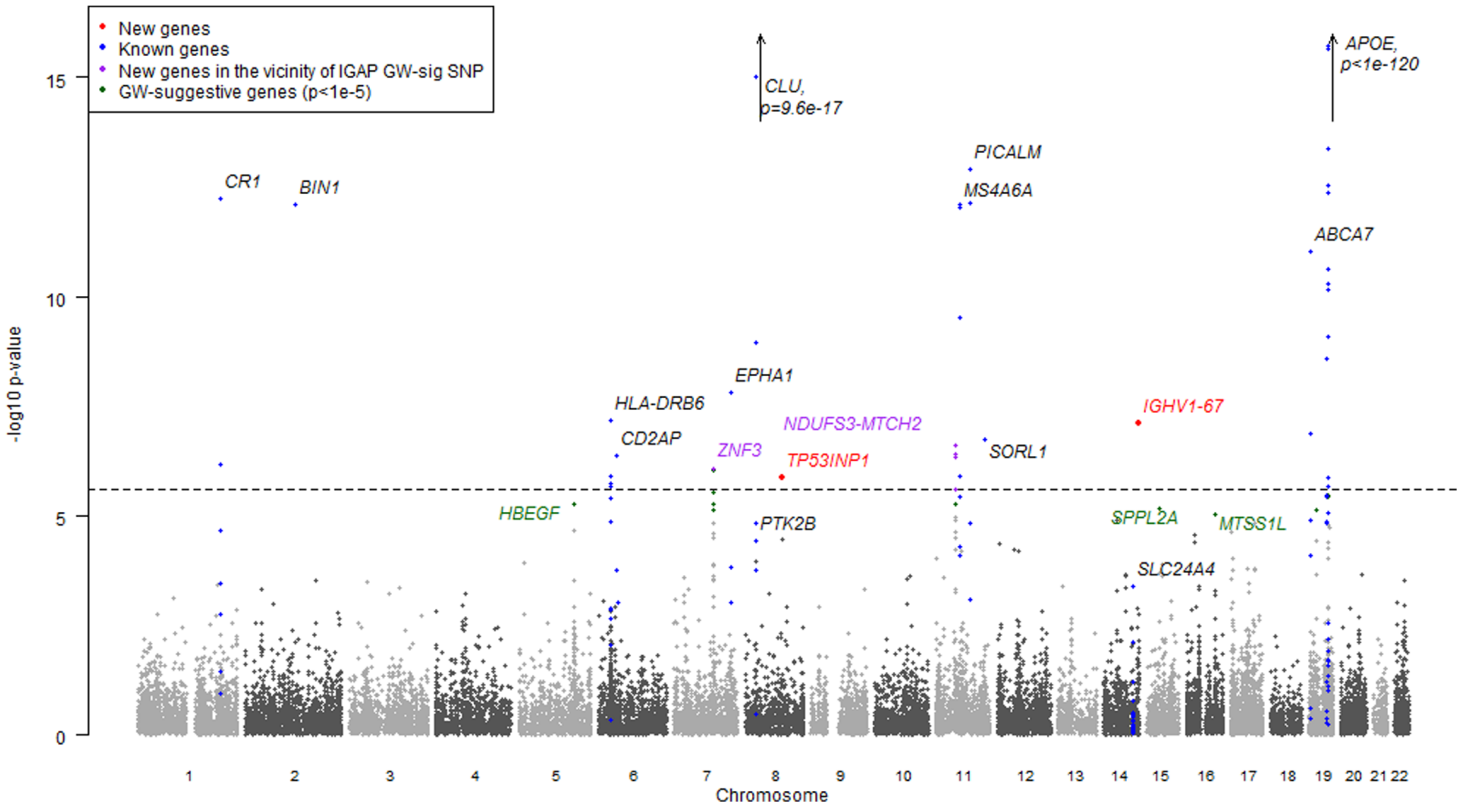
Manhattan plot of gene-wide p-values in the Stage 1 dataset and combined gene-wide p-values where Stage 2 data are available. Each dot represents a gene, genes in blue lie within the previously reported[4]–[8] associated regions.

The Manhattan plot of the gene-based p-values (Figure 3) gives a general overview of the gene-based results and shows the new loci in relation to previously reported genes (see also QQ-plots in Figure S4). The results of gene-wide analysis for the genes, which were previously reported as associated with AD[4]-[8] and those which are GWAS significant in the Stage 1 analysis are presented in Table S9. Out of 16 reported susceptibility genes, 15 are nominally significant with gene-wide analysis (almost all p-values are smaller than 10^−4^), however not all of them reach the gene-based genome-wide significance level (2.5×10^−6^) when the number of SNPs per gene and LD structure of the gene is taken into account.

We did not observe genome-wide significance for *CD33* gene. This gene was genome-wide significant in Stage 1 (p = 1.9×10^−6^), but the association was attenuated when combining Stage 1 and Stage 2 data (p = 1.79×10^−5^), similar to the single SNP association result in the SNP GWAS study[9], [19].

## Discussion

In this study we show that there are more signals in the GWAS imputed data at SNP- and gene-based levels than revealed by single SNP analysis. A gene-based analysis is a next logical step after the single SNP analyses in any attempt to combine possible several signals in genes and thus enhance the power of the association analyses.

The first new gene *TP53INP1* (chromosome 8) encodes a protein that is involved in mediating autophagy-dependent cell death via apoptosis through altering the phosphorylation state of p53[23] and in modulating cell-extracellular matrix adhesion and cell migration[24]. *TP53INP1* encodes a pro-apoptotic tumor suppressor and its antisense oligonucleotide has been used as potential treatment for castration-resistant prostate cancer[25]. This association is notable, given the potential inverse association between cancer and AD that has previously been reported [26], [27].

The second new gene *IGHV1-67* (chromosome 14) is a pseudogene in the immunoglobulin (IgG) variable heavy chain region of chromosome 14: its function is unknown but all genes in this region are most likely to be involved in IgG heavy chain VDJ recombinations that lead to the full repertoire of antigen-detecting immune cell clones[28].

The gene-based analysis in this study has shown its utility to enhance the information provided by single SNP analysis (i.e. *NDUFS3* gene was genome-wide significant from Stage 1 using gene-based analysis whereas this gene was only genome-wide significant after combining the two stages of single SNP analysis).

*ZNF3* is a zinc-finger protein at the same locus on chromosome 7 as *ZCWPW1* thus rendering it a candidate as the gene that contains the functional signal in this region. Although we can not identify which gene actually confers the risk to AD, it is interesting that *ZNF3* function is unknown though it interacts with BAG3 which is involved in ubiquitin/proteasomal functions in protein degradation[29] and *ZNF3* is regulated by upstream binding of BACH1 whose target genes have roles in the oxidative stress response and control of the cell cycle[30].

In the cluster of genes on chromosome 11, *MTCH2* encodes one of the large family of inner mitochondrial membrane transporters[31] which is associated with mitochondrially-mediated cell death[32], adipocyte differentiation[33], insulin sensitivity[34] and has a genetic association with increased BMI[35]. *NDUFS3* also has functions in the mitochondria as it encodes an iron-sulphur component of complex 1 (mitochondrial NADH:ubiquinone oxidoreductase) of the electron transport chain. A deficiency causes a form of Leigh syndrome[36] an early-onset progressive neurodegenerative disorder with a characteristic neuropathology consisting of focal lesions including areas of demyelination and gliosis[37].

In summary, we report two novel genes *TP53INP1* (chr8: 95,938,200–95,961,615; combined p = 1.4×10^−6^) and *IGHV1-67* (chr14: 107,136,620–107,137,059; combined p = 7.9×10^−8^), which were not reported as genome-wide significant before. We also report *ZNF3* gene on chromosome 7 and a cluster of genes on chromosome 11 (*SPI1*-*MTCH2*), showing gene-based genome-wide significant association with Alzheimer's disease. These genes are in proximity with, but not the same as, those detected by genome-wide significant SNPs, demonstrating support for the signals identified by IGAP[9], [19]. They have an array of functions previously implicated in AD including aspects of energy metabolism, protein degradation and the immune system and add further weight to these pathways as potential therapeutic targets in AD.

## Materials and Methods

### Stage 1 data

The main dataset was reported by the IGAP consortium[9], [19] and consists in total of 17,008 cases and 37,154 controls. This sample of AD cases and controls comprises 4 data sets taken from genome-wide association studies performed by GERAD, EADI, CHARGE and ADGC (see primary IGAP manuscript[9], [19] for more details). The full details of the samples and methods for conduct of the GWA studies are provided in the respective manuscripts[4]-[8].

Each of these datasets was imputed with Impute2[38] or MACH[39] software using the 1000 genomes data (release Dec2010) as a reference panel. In total 11,863,202 SNPs were included in the SNPs allelic association result file. To make our analysis as conservative as possible, we only included autosomal SNPs which passed stringent quality control criteria, i.e. we included only SNPs with minor allele frequencies (MAF) ≥0.01 and imputation quality score greater than or equal to 0.3 in each individual study, resulting in 7,055,881 SNPs which are present in at least 40% of the AD cases and 40% of the controls in the analysis. The summary statistics across datasets were combined using fixed-effects inverse variance-weighted meta-analysis. We corrected all individual SNPs p-values for genomic control (GC) λ = 1.087. These SNPs are well imputed on a large proportion of the sample, which increases confidence in the accuracy of the association analysis upon which gene-wide analysis is based.

### Stage 2 data

11,632 SNPs with p-values <10^−3^ in the IGAP meta-analysis were successfully genotyped in a Stage 2 sample comprising 8,572 cases and 11,312 controls (see primary IGAP manuscript[9], [19] for more details). An additional 771 SNPs were successfully genotyped to test all genes with gene-wide p-values <10^-4^ in the IGAP Stage 1 analysis, excluding genes reported prior to IGAP[4]–[8], the four loci reaching genome-wide significance in the Stage 1 IGAP meta-analysis[9], [19] and the 0.5Mb regions around them (Table S2). These SNPs cover 887 genes and correspond to 444 independent loci where all genes within 0.5 Mb are counted as one locus.

### Assignment of SNPs to genes

SNPs were assigned to genes if they were located within the genomic sequence lying between the start of the first and the end of the last exon of any transcript corresponding to that gene. The chromosome and location for all currently known human SNPs were taken from the dbSNP132 database, as was their assignment to genes (using build 37.1). In total, we retained 2,804,431 (39.7% of the total) SNPs which annotated 28,636 unique genes with 1–16,514 SNPs per gene. For the gene-wide analysis we have excluded genes which contain only one SNP in the IGAP Stage 1 analysis, leaving a total of 25,310 genes. If a SNP belongs to more than one gene, it was assigned to each of these genes. In order to account for possible signals which are correlated with those in a gene, gene-wide analysis was also performed using a 10 kb window around genes to assign SNPs to genes.

### Gene-wide analysis

The gene-wide analysis was performed based on the summary p-values while controlling for LD and different number of markers per gene using an approximate statistical approach[40] adopted for set-based analysis of genetic data[41]. This is a method for calculating the significance of a set of SNPs in the absence of individual genotype data based on a theoretical approximation to Fisher's statistic for combining p-values. Fisher's statistic (-∑ln(p_i_)) combines probabilities and under the null hypothesis has a chi-square distribution with 2N degrees of freedom, where N is the number of markers, and the summation above is for *i*  = 1,…,N). If Fisher's statistic combines the results of several tests when the tests are independent, the approximate method combines non-independent tests and requires only the list of p-values for each SNP and knowledge of correlations between SNPs. Then the value of Fisher's statistic and the number of degrees of freedom is corrected by the coefficient which depends upon the number of SNPs and correlations (LD) between them. This approximation was applied to the Stage 1 and Stage 2 samples separately, and the resulting gene-wide p-values combined using Fisher's method (since these are independent). LD between markers was computed using 1000 genomes data. The gene-based genome-wide significant level was set to 2.5×10^−6^ to account for the number of tested genes[42].

### Test for excess of associated SNPs/loci

The effective number *N* of independent SNPs in the whole genome (excluding genes with SNPs that are genome-wide significant in the Stage 1 IGAP dataset ± 0.5 Mb was estimated by the method described in [43] taking LD into account, as were the observed number of independent SNPs significant at each p-value criterion (adjusting individual SNP p-values for genomic control λ = 1.087 before hand). LD was computed from the 1000 Genomes database (http://www.1000genomes.org/). In the absence of excess association, the expected number of independent SNPs significant at significance level α is a normally distributed random variable whose mean and standard deviation (SD) can be calculated as *αN* and √*Nα*(1-*α*) (mean and SD for a binomial distribution). The number of independent SNPs (and thus statistical tests) in the whole genome were estimated as ∼3.7×10^6^, ∼3.6×10^6^ and ∼3.5×10^6^ at significance levels below 0.1, between 0.05 and 0.1, and 0.2 and above respectively (see [43] for details on the dependence between the significance levels and the estimated number of independent tests). We then calculated mean of the expected number of significant SNPs in intervals *α*_1_ < p ≤ *α*_2_, (*α*_1_, *α*_2_  = 0, 10^−6^, 10^−5^, …, 0.5) as difference between the expected numbers of independent SNPs at *α*_2_ and *α*_1_ significance levels and SD as the square root of sum of the corresponding variances.

We calculated the significance of the excess *number* of genes attaining the specified thresholds based upon the assumption that, under the null hypothesis of no association, the number of significant genes at a significance level of *α* in a scan is distributed as a binomial (*N,α*), where *N* is the total number of genes, assuming that genes are independent. Genes within 0.5 Mb of each other are counted as one signal when calculating the observed number of *significant* genes. This prevents significance being inflated by LD between genes, where a single association signal gives rise to several significantly-associated genes. The *total* number of genes was not corrected for LD in this way, making the estimate of significance of the excess *number* of genes conservative.

## Supporting Information

Table S1 **Overrepresentation of significant SNPs excluding previously reported** [4]-[8] **genes ±0.5Mb and the APOE region as above.**
(DOCX)

Table S2 **List of genes that are genome-wide significant in the IGAP stage 1 dataset and the flanking regions which included SNPs either in r^2^≥0.3 or association p-value≤10^-3^ whichever covers the largest region.**
(DOCX)

Table S3 **Detailed SNP information for TP53INP1 gene.**
(XLS)

Table S4 **Detailed SNP information for IGHV1-67 gene.**
(XLS)

Table S5 **Detailed SNP information for ZNF3 gene.**
(XLS)

Table S6 **Detailed SNP information for NDUFS3 gene.**
(XLS)

Table S7 **Detailed SNP information for MTCH2 gene.**
(XLS)

Table S8 **Gene-based analysis results, when single SNPs p-values, contributing to the gene-based p-value were adjusted for the best genome-wide significant SNP in the nearby location.**
(DOCX)

Table S9 **Gene-wide analysis for genes which show GWAS significant association with AD in the stage 1 IGAP dataset.**
(DOCX)

Table S10 **Brain eQTL Tissues.**
(XLSX)

Table S11 **Brain Meth QTLs.**
(XLSX)

Figure S1 ***ZNF3* gene with rs1476679 (*ZCWPW1*) reported by Lambert et al (2013) study.** SNPs which are significant at 1e-3 level are circled in red, rs1476679 is highlighted in blue.
(TIF)

Figure S2 ***NDUFS3* gene rs10838725 (*CELF1*) reported by Lambert et al (2013) study.** SNPs which are significant at 1e-3 level are circled in red, rs10838725 is highlighted in blue.
(TIF)

Figure S3 ***MTCH2* gene with rs10838725 (*CELF1*) reported by Lambert et al (2013) study.** SNPs which are significant at 1e-3 level are circled in red, rs10838725 is highlighted in blue.
(TIF)

Figure S4 **QQ-plot of gene-wide p-values for all genes (A) and excluding previously reported** [4]-[8] **GWAS significantly associated genes ±0.5Mb (B) in the discovery dataset.** Genomic control λ = 1.08 and 1.07 respectively.
(TIFF)

Methods S1 **Expression quantitative trait loci (eQTL) and Methylation quantitative trait loci (meQTL) analyses.**
(DOCX)

Materials S1 **Full IGAP datasets description.**
(DOCX)

Materials S2 **List of IGAP consortium members.**
(DOC)

Materials S3 **Acknowledgements.**
(DOCX)

## References

[1] Gatz M, Reynolds CA, Fratiglioni L, Johansson B, Mortimer JA, et al (2006) Role of genes and environments for explaining Alzheimer disease. Archives of General Psychiatry 63: 168–174. 16461860 10.1001/archpsyc.63.2.168

[2] Bettens K, Sleegers K, Van Broeckhoven C (2013) Genetic insights in Alzheimer's disease. Lancet neurology 12: 92–104. 23237904 10.1016/S1474-4422(12)70259-4

[3] Corder EH, Saunders AM, Strittmatter WJ, Schmechel DE, Gaskell PC, et al (1993) Gene dose of apolipoprotein E type 4 allele and the risk of Alzheimer's disease in late onset families. Science 261: 921–923. 8346443 10.1126/science.8346443

[4] Harold D, Abraham R, Hollingworth P, Sims R, Gerrish A, et al (2009) Genome-wide association study identifies variants at CLU and PICALM associated with Alzheimer's disease. Nature genetics 41: 1088–1093. 19734902 10.1038/ng.440 PMC2845877

[5] Hollingworth P, Harold D, Sims R, Gerrish A, Lambert JC, et al (2011) Common variants at ABCA7, MS4A6A/MS4A4E, EPHA1, CD33 and CD2AP are associated with Alzheimer's disease. Nature Genetics 43: 429–435. 21460840 10.1038/ng.803 PMC3084173

[6] Lambert JC, Heath S, Even G, Campion D, Sleegers K, et al (2009) Genome-wide association study identifies variants at CLU and CR1 associated with Alzheimer's disease. Nature Genetics 41: 1094–U1068. 19734903 10.1038/ng.439

[7] Naj AC, Jun G, Beecham GW, Wang LS, Vardarajan BN, et al (2011) Common variants at MS4A4/MS4A6E, CD2AP, CD33 and EPHA1 are associated with late-onset Alzheimer's disease. Nature Genetics 43: 436–441. 21460841 10.1038/ng.801 PMC3090745

[8] Seshadri S, Fitzpatrick AL, Ikram MA, DeStefano AL, Gudnason V, et al (2010) Genome-wide analysis of genetic loci associated with Alzheimer disease. JAMA: the journal of the American Medical Association 303: 1832–1840. 20460622 10.1001/jama.2010.574 PMC2989531

[9] Lambert JC, Ibrahim-Verbaas CA, Harold D, Naj AC, Sims R, et al (2013) Meta-analysis of 74,046 individuals identifies 11 new susceptibility loci for Alzheimer's disease. Nat Genet 45: 1452–1458. 24162737 10.1038/ng.2802 PMC3896259

[10] Guerreiro RJ, Hardy J (2011) Alzheimer's disease genetics: lessons to improve disease modelling. Biochemical Society transactions 39: 910–916. 21787322 10.1042/BST0390910

[11] Ioannidis JP (2007) Non-replication and inconsistency in the genome-wide association setting. Human heredity 64: 203–213. 17551261 10.1159/000103512

[12] Neale BM, Sham PC (2004) The future of association studies: gene-based analysis and replication. American journal of human genetics 75: 353–362. 15272419 10.1086/423901 PMC1182015

[13] Moskvina V, O'Donovan MC (2007) Detailed analysis of the relative power of direct and indirect association studies and the implications for their interpretation. Human heredity 64: 63–73. 17483598 10.1159/000101424

[14] Terwilliger JD, Hiekkalinna T (2006) An utter refutation of the "fundamental theorem of the HapMap". European journal of human genetics: EJHG 14: 426–437. 16479260 10.1038/sj.ejhg.5201583

[15] Bettens K, Brouwers N, Engelborghs S, Lambert JC, Rogaeva E, et al (2012) Both common variations and rare non-synonymous substitutions and small insertion/deletions in CLU are associated with increased Alzheimer risk. Molecular neurodegeneration 7: 3. 22248099 10.1186/1750-1326-7-3 PMC3296573

[16] Risch N (1990) Linkage strategies for genetically complex traits. I. Multilocus models. American journal of human genetics 46: 222–228. 2301392 PMC1684987

[17] Lewis CM, Levinson DF, Wise LH, DeLisi LE, Straub RE, et al (2003) Genome scan meta-analysis of schizophrenia and bipolar disorder, part II: Schizophrenia. American journal of human genetics 73: 34–48. 12802786 10.1086/376549 PMC1180588

[18] Segurado R, Detera-Wadleigh SD, Levinson DF, Lewis CM, Gill M, et al (2003) Genome scan meta-analysis of schizophrenia and bipolar disorder, part III: Bipolar disorder. American journal of human genetics 73: 49–62. 12802785 10.1086/376547 PMC1180589

[19] Lambert JCea (2013) Extended meta-analysis of 74,538 individuals identifies 11 new susceptibility loci for Alzheimer's disease. 10.1038/ng.2802 PMC3896259 24162737

[20] Johnson AD, Handsaker RE, Pulit SL, Nizzari MM, O'Donnell CJ, et al (2008) SNAP: a web-based tool for identification and annotation of proxy SNPs using HapMap. Bioinformatics 24: 2938–2939. 18974171 10.1093/bioinformatics/btn564 PMC2720775

[21] Webster JA, Gibbs JR, Clarke J, Ray M, Zhang WX, et al (2009) Genetic Control of Human Brain Transcript Expression in Alzheimer Disease. American Journal of Human Genetics 84: 445–458. 19361613 10.1016/j.ajhg.2009.03.011 PMC2667989

[22] Trabzuni D, Ryten M, Walker R, Smith C, Imran S, et al (2011) Quality control parameters on a large dataset of regionally dissected human control brains for whole genome expression studies. J Neurochem 119: 275–282. 21848658 10.1111/j.1471-4159.2011.07432.x PMC3664422

[23] Seux M, Peuget S, Montero MP, Siret C, Rigot V, et al (2011) TP53INP1 decreases pancreatic cancer cell migration by regulating SPARC expression. Oncogene 30: 3049–3061. 21339733 10.1038/onc.2011.25

[24] Seillier M, Peuget S, Gayet O, Gauthier C, N'Guessan P, et al (2012) TP53INP1, a tumor suppressor, interacts with LC3 and ATG8-family proteins through the LC3-interacting region (LIR) and promotes autophagy-dependent cell death. Cell death and differentiation 19: 1525–1535. 22421968 10.1038/cdd.2012.30 PMC3422476

[25] Giusiano S, Baylot V, Andrieu C, Fazli L, Gleave M, et al (2012) TP53INP1 as new therapeutic target in castration-resistant prostate cancer. Prostate 72: 1286–1294. 22213058 10.1002/pros.22477

[26] Driver JA, Beiser A, Au R, Kreger BE, Splansky GL, et al.. (2012) Inverse association between cancer and Alzheimer's disease: results from the Framingham Heart Study. British Medical Journal 344. 10.1136/bmj.e1442 PMC3647385 22411920

[27] Roe CM, Fitzpatrick AL, Xiong C, Sieh W, Kuller L, et al (2010) Cancer linked to Alzheimer disease but not vascular dementia. Neurology 74: 106–112. 20032288 10.1212/WNL.0b013e3181c91873 PMC2809029

[28] Watson CT, Breden F (2012) The immunoglobulin heavy chain locus: genetic variation, missing data, and implications for human disease. Genes and immunity 13: 363–373. 22551722 10.1038/gene.2012.12

[29] Chen Y, Yang LN, Cheng L, Tu S, Guo SJ, et al.. (2013) BAG3 Interactome Analysis Reveals a New Role in Modulating Proteasome Activity. Molecular & cellular proteomics: MCP. 10.1074/mcp.M112.025882 PMC3790292 23824909

[30] Warnatz HJ, Schmidt D, Manke T, Piccini I, Sultan M, et al (2011) The BTB and CNC homology 1 (BACH1) target genes are involved in the oxidative stress response and in control of the cell cycle. The Journal of biological chemistry 286: 23521–23532. 21555518 10.1074/jbc.M111.220178 PMC3123115

[31] Palmieri F (2013) The mitochondrial transporter family SLC25: identification, properties and physiopathology. Molecular aspects of medicine 34: 465–484. 23266187 10.1016/j.mam.2012.05.005

[32] Katz C, Zaltsman-Amir Y, Mostizky Y, Kollet N, Gross A, et al (2012) Molecular basis of the interaction between proapoptotic truncated BID (tBID) protein and mitochondrial carrier homologue 2 (MTCH2) protein: key players in mitochondrial death pathway. The Journal of biological chemistry 287: 15016–15023. 22416135 10.1074/jbc.M111.328377 PMC3340256

[33] Bernhard F, Landgraf K, Kloting N, Berthold A, Buttner P, et al (2013) Functional relevance of genes implicated by obesity genome-wide association study signals for human adipocyte biology. Diabetologia 56: 311–322. 23229156 10.1007/s00125-012-2773-0

[34] Fall T, Arnlov J, Berne C, Ingelsson E (2012) The role of obesity-related genetic loci in insulin sensitivity. Diabetic medicine: a journal of the British Diabetic Association 29: e62–66. 22443470 10.1111/j.1464-5491.2012.03665.x

[35] Haupt A, Thamer C, Heni M, Machicao F, Machann J, et al (2010) Novel obesity risk loci do not determine distribution of body fat depots: a whole-body MRI/MRS study. Obesity 18: 1212–1217. 19910938 10.1038/oby.2009.413

[36] Benit P, Slama A, Cartault F, Giurgea I, Chretien D, et al (2004) Mutant NDUFS3 subunit of mitochondrial complex I causes Leigh syndrome. Journal of medical genetics 41: 14–17. 14729820 10.1136/jmg.2003.014316 PMC1757256

[37] Dahl HH (1998) Getting to the nucleus of mitochondrial disorders: identification of respiratory chain-enzyme genes causing Leigh syndrome. American journal of human genetics 63: 1594–1597. 9837811 10.1086/302169 PMC1377630

[38] Howie BN, Donnelly P, Marchini J (2009) A flexible and accurate genotype imputation method for the next generation of genome-wide association studies. PLoS genetics 5: e1000529. 19543373 10.1371/journal.pgen.1000529 PMC2689936

[39] Li Y, Willer CJ, Ding J, Scheet P, Abecasis GR (2010) MaCH: using sequence and genotype data to estimate haplotypes and unobserved genotypes. Genetic epidemiology 34: 816–834. 21058334 10.1002/gepi.20533 PMC3175618

[40] Brown MB (1975) A method for combining non-independent, one-sided tests of significance. Biometrics 31: 978–992.

[41] Moskvina V, O'Dushlaine C, Purcell S, Craddock N, Holmans P, et al (2011) Evaluation of an approximation method for assessment of overall significance of multiple-dependent tests in a genomewide association study. Genetic epidemiology 35: 861–866. 22006681 10.1002/gepi.20636 PMC3268180

[42] Kiezun A, Garimella K, Do R, Stitziel NO, Neale BM, et al (2012) Exome sequencing and the genetic basis of complex traits. Nature genetics 44: 623–630. 22641211 10.1038/ng.2303 PMC3727622

[43] Moskvina V, Schmidt KM (2008) On multiple-testing correction in genome-wide association studies. Genetic epidemiology 32: 567–573. 18425821 10.1002/gepi.20331

