# Supplementary material for: Gene-Wide Analysis Detects Two New Susceptibility Genes for Alzheimer's Disease
Source: PLoS One. 2014 Jun 12;9(6):e94661. doi: 10.1371/journal.pone.0094661 (PMC4055488; doi:10.1371/journal.pone.0094661)
Supplement: Table S1 — Overrepresentation of significant SNPs excluding previously reported [4]-[8] genes ±0.5Mb and the APOE region as above. (DOCX) [file pone.0094661.s001.docx]

**Table S1.** Overrepresentation of significant SNPs excluding previously reported[^5-9^](#_ENREF_5) genes ± 0.5Mb and the APOE region as above.

| interval α | N SNPs | Est. N of independent sig. SNPs | Expected  N SNPs (mean±SD) | Ratio Est/Exp | p-value |
| --- | --- | --- | --- | --- | --- |
|  |  |  |  |  |  |
| 0 < p ≤ 10^-6^ | 9 | 8 | 4±2.0 | 2.03 | 0.015 |
| 10^-6^ < p ≤ 10^-5^ | 133 | 61 | 33±6.4 | 1.83 | 1.6x10^-5^ |
| 10^-5^ < p ≤ 10^-4^ | 1515 | 868 | 334±20 | 2.20 | 2.8x10^-154^ |
| 10^-4^ < p ≤ 10^-3^ | 8562 | 5044 | 3339±64 | 1.51 | 3.0x10^-157^ |
| 10^-3^ < p ≤ 0.01 | 66524 | 36772 | 33386±201 | 1.10 | 1.3x10^-63^ |
| 0.01 < p ≤ 0.1 | 630948 | 330229 | 324707±602 | 1.02 | 4.6x10^-20^ |
| 0.1 < p ≤ 0.2 | 698604 | 370270 | 347297±945 | 1.07 | 1.4x10^-130^ |
| 0.2 < p ≤ 0.3 | 692171 | 382472 | 347330±1143 | 1.10 | 1.6x10^-207^ |
| 0.3 < p ≤ 0.4 | 696887 | 393565 | 345110±1257 | 1.14 | <10^-256^ |
| 0.4 < p ≤ 0.5 | 701195 | 401522 | 350385±1310 | 1.15 | <10^-256^ |

Exp = expected, signif = significant. SD = standard deviation, Est = estimated number[^21^](#_ENREF_21) of independent SNPs. The number of significant SNPs in the intervals are counted while adjusting individual SNP p-values for genomic control λ=1.087.
