# Supplementary material for: Gene-Wide Analysis Detects Two New Susceptibility Genes for Alzheimer's Disease
Source: PLoS One. 2014 Jun 12;9(6):e94661. doi: 10.1371/journal.pone.0094661 (PMC4055488; doi:10.1371/journal.pone.0094661)
Supplement: Table S2 — List of genes that are genome-wide significant in the IGAP stage 1 dataset and the flanking regions which included SNPs either in r2≥0.3 or association p-value≤10-3 whichever covers the largest region. (DOCX) [file pone.0094661.s002.docx]

**Table S2.** List of genes that are genome-wide significant in the IGAP stage 1 dataset and the flanking regions which included SNPs either in r^2^≥0.3 or association p-value≤10^-3^ whichever covers the largest region.

|  | GENE | CHR | 5’ | 3’ |
| --- | --- | --- | --- | --- |
| 1 | *CR1* | 1 | 207,626,000 | 207,926,000 |
| 2 | *BIN1* | 2 | 127,770,000 | 127,900,000 |
| 3 | *CD2AP* | 6 | 47,310,000 | 47,680,000 |
| 4 | *HLA-DRB5/HLA-DRB1* | 6 | 32,200,000 | 32,700,000 |
| 5 | *EPHA1* | 7 | 143,068,204 | 143,145,000 |
| 6 | *PTK2B* | 8 | 27,163,054 | 27,336,905 |
| 7 | *CLU* | 8 | 27,434,450 | 27,505,000 |
| 8 | *MS4A6A* | 11 | 59,820,000 | 60,130,000 |
| 9 | *PICALM* | 11 | 85,615,000 | 85,905,000 |
| 10 | *SORL1* | 11 | 121,302,960 | 121,600,000 |
| 11 | *SLC24A4/RIN3* | 14 | 92,000,000 | 94,000,000 |
| 12 | *ABCA7* | 19 | 1,010,000 | 1,095,000 |
| 13 | *APOE* | 19 | 44,911,940 | 45,911,945 |
