# Supplementary material for: Gene-Wide Analysis Detects Two New Susceptibility Genes for Alzheimer's Disease
Source: PLoS One. 2014 Jun 12;9(6):e94661. doi: 10.1371/journal.pone.0094661 (PMC4055488; doi:10.1371/journal.pone.0094661)
Supplement: Table S8 — Gene-based analysis results, when single SNPs p-values, contributing to the gene-based p-value were adjusted for the best genome-wide significant SNP in the nearby location. (DOCX) [file pone.0094661.s008.docx]

**Table S8.** Gene-based analysis results, when single SNPs p-values, contributing to the gene-based p-value were adjusted for the best genome-wide significant SNP in the nearby location.

| Gene Name | Chr | Position | Combined  gene-wide  p-value | Single SNP genome-wide significant hit | Single SNP  genome-wide  significant hit combined  Stage1-Stage2 p-value | Gene-based p-value conditioning for the genome-wide  significant hit |
| --- | --- | --- | --- | --- | --- | --- |
| *ZNF3* | 7 | 99,661,653-99,679,371 | 8.6x10^-7^ | rs1476679  (100,004,446) | 5.6x10^-10^ | 0.347 |
| *NDUFS3* | 11 | 47,600,632-47,606,114 | 4.8x10^-7^ | rs10838725  (47,557,871) | 1.1x10^-8^ | 0.081 |
| *MTCH2* | 11 | 47,638,858-47,664,206 | 2.5x10^-6^ | rs10838725  (47,557,871) | 1.1x10^-8^ | 0.203 |
