# Supplementary material for: Gene-Wide Analysis Detects Two New Susceptibility Genes for Alzheimer's Disease
Source: PLoS One. 2014 Jun 12;9(6):e94661. doi: 10.1371/journal.pone.0094661 (PMC4055488; doi:10.1371/journal.pone.0094661)
Supplement: Table S9 — Gene-wide analysis for genes which show GWAS significant association with AD in the stage 1 IGAP dataset. (DOCX) [file pone.0094661.s009.docx]

**Table S9.** Gene-wide analysis for genes which show GWAS significant association with AD in the stage 1 IGAP dataset.

| Gene Name | Chr | Positions | | N SNPs | Gene-wide  p-value | N SNPs  with p≤0.05 | Best SNP  p-value |
| --- | --- | --- | --- | --- | --- | --- | --- |
|  |  | Start SNP | End SNP |  |  |  |  |
| *CR1* | 1 | 207,669,473 | 207,815,110 | 251 | 3.5x10^-7^ | 124 | 7.7x10^-15^ |
| *BIN1* | 2 | 127,805,607 | 127,864,864 | 220 | 4.8x10^-6^ | 134 | 3.7x10^-16^ |
| *HLA-DRB1* | 6 | 32,546,546 | 32,557,562 | 595 | 6.6x10^-6^ | 307 | 1.6x10^-8^ |
| *CD2AP* | 6 | 47,445,525 | 47,594,999 | 319 | 8.0x10^-6^ | 257 | 3.1x10^-8^ |
| *EPHA1* | 7 | 143,088,205 | 143,105,985 | 39 | 3.9x10^-7^ | 15 | 3.4x10^-11^ |
| *PTK2B* | 8 | 27,168,999 | 27,316,903 | 380 | 1.3x10^-4^ | 188 | 3.3x10^-9^ |
| *CLU* | 8 | 27,454,451 | 27,472,327 | 35 | 5.1x10^-15^ | 30 | 9.6x10^-17^ |
| *MS4A6A* | 11 | 59,939,080 | 59,950,674 | 22 | 3.7x10^-10^ | 13 | 1.6x10^-10^ |
| *PICALM* | 11 | 85,668,485 | 85,780,108 | 302 | 1.2x10^-8^ | 243 | 1.9x10^-12^ |
| *SORL1* | 11 | 121,322,961 | 121,504,471 | 319 | 2.7x10^-5^ | 94 | 5.0x10^-11^ |
| *SLC24A4* | 14 | 92,788,925 | 92,962,596 | 675 | 0.0001 | 242 | 1.5x10^-7^ |
| *DSG2* | 18 | 29,078,027 | 29,128,814 | 122 | 0.593 | 6 | 4.6x10^-8^ |
| *ABCA7* | 19 | 1,040,102 | 1,065,571 | 93 | 3.0x10^-7^ | 60 | 1.7x10^-9^ |
| *APOE* | 19 | 45,409,039 | 45,412,650 | 5 | <10^-200^ | 5 | 5.5x10^-25^ |
| *CD33* | 19 | 51,728,335 | 51,743,274 | 23 | 1.9x10^-6^ | 11 | 6.5x10^-8^ |
