# Supplementary figures and images for: Gene-Wide Analysis Detects Two New Susceptibility Genes for Alzheimer's Disease
Source: PLoS One. 2014 Jun 12;9(6):e94661. doi: 10.1371/journal.pone.0094661 (PMC4055488; doi:10.1371/journal.pone.0094661)

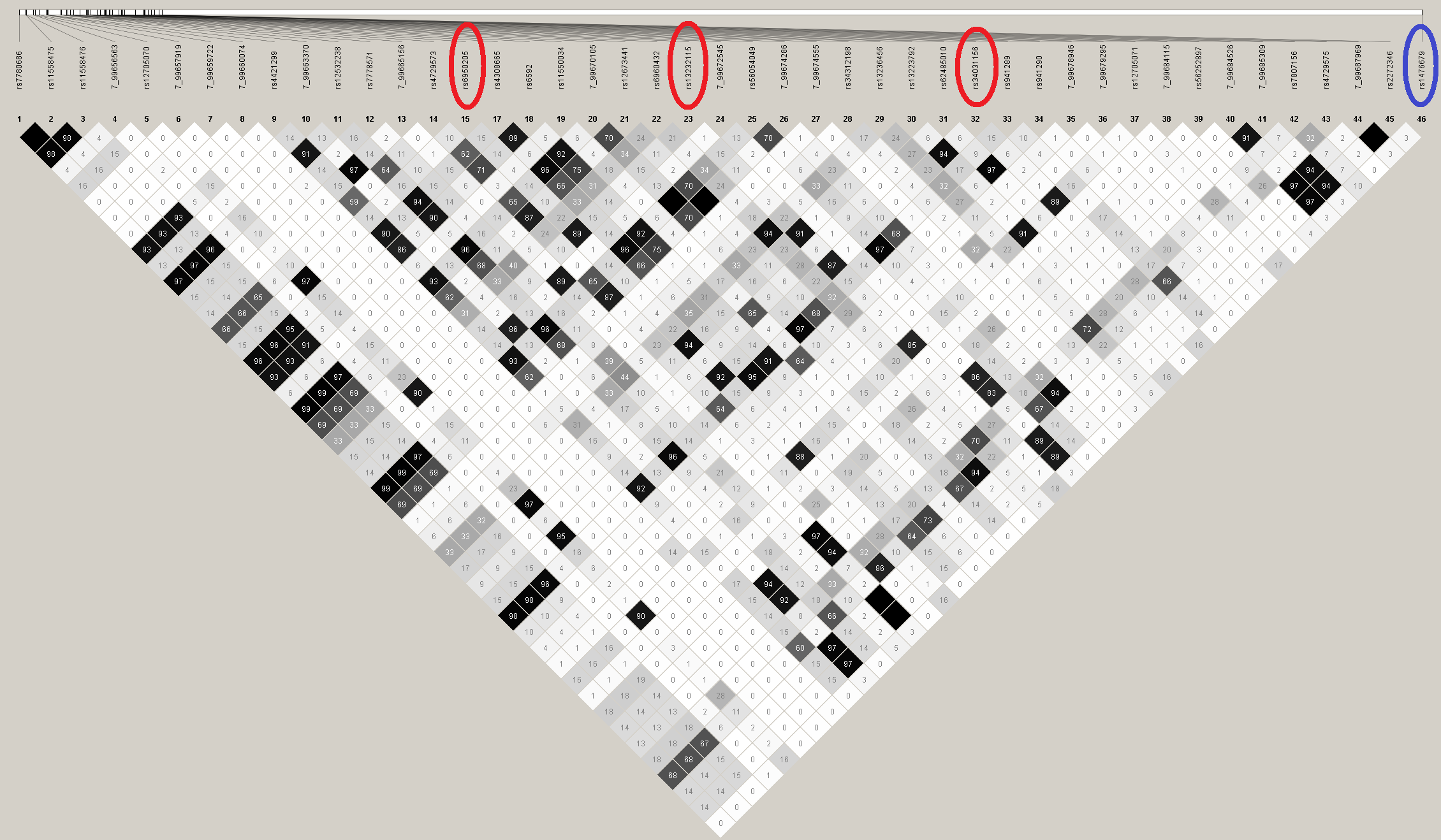

Supplement: Figure S1 — ZNF3 gene with rs1476679 (ZCWPW1) reported by Lambert et al (2013) study. SNPs which are significant at 1e-3 level are circled in red, rs1476679 is highlighted in blue. (TIF) [file pone.0094661.s012.tif]

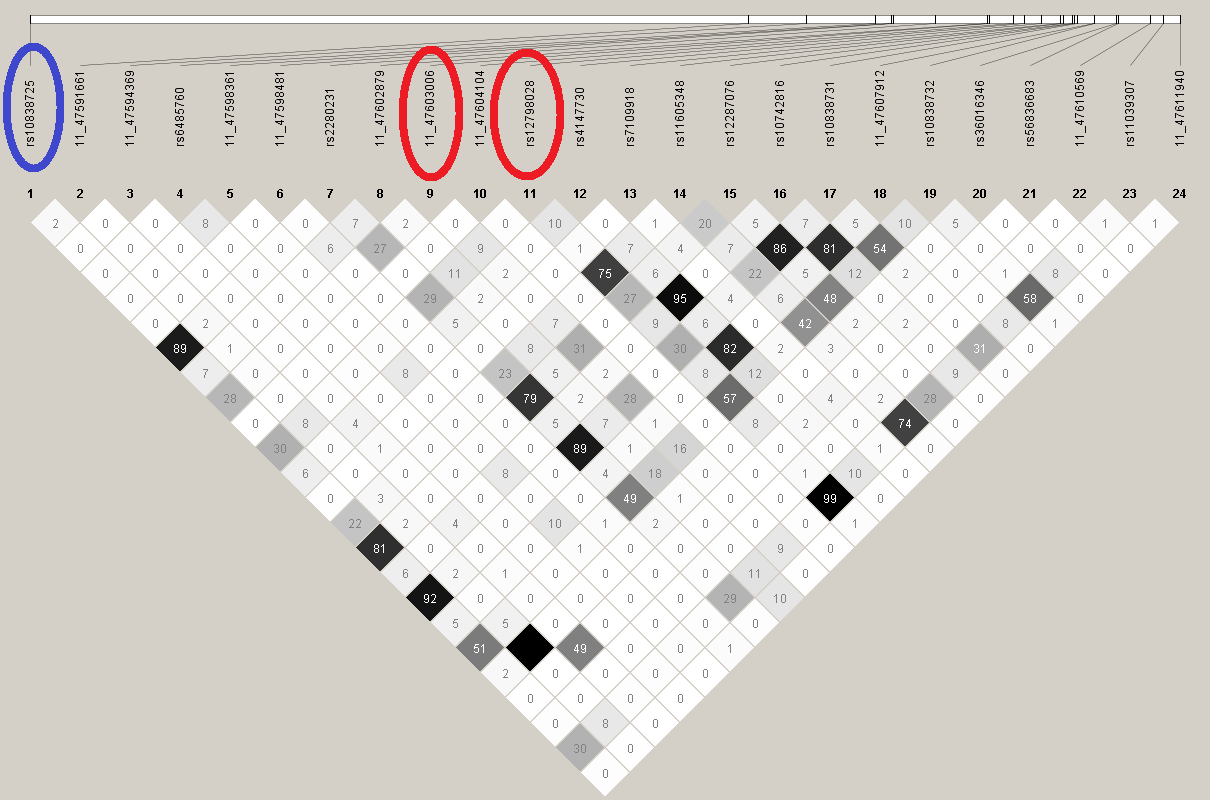

Supplement: Figure S2 — NDUFS3 gene rs10838725 (CELF1) reported by Lambert et al (2013) study. SNPs which are significant at 1e-3 level are circled in red, rs10838725 is highlighted in blue. (TIF) [file pone.0094661.s013.tif]

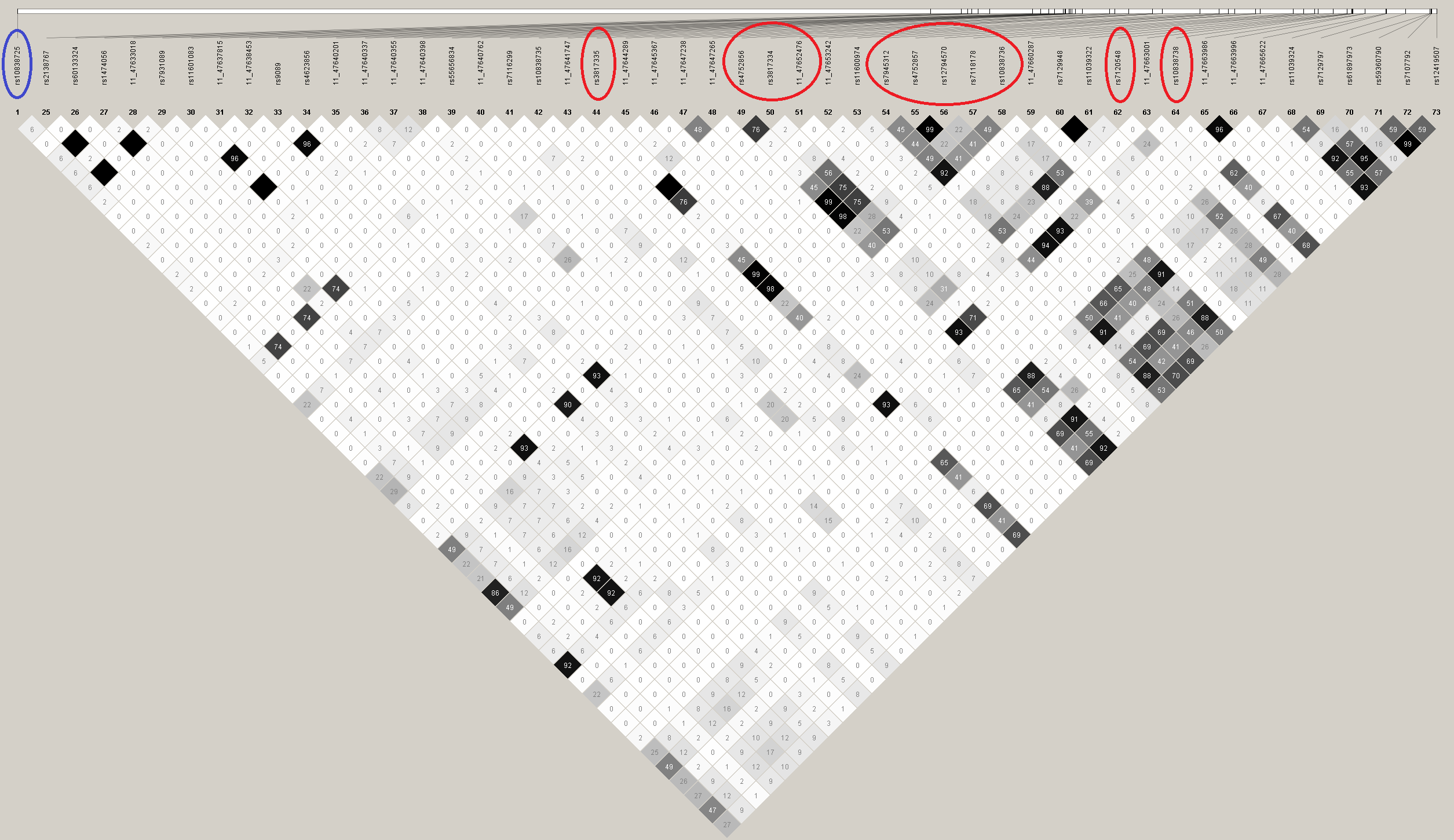

Supplement: Figure S3 — MTCH2 gene with rs10838725 (CELF1) reported by Lambert et al (2013) study. SNPs which are significant at 1e-3 level are circled in red, rs10838725 is highlighted in blue. (TIF) [file pone.0094661.s014.tif]

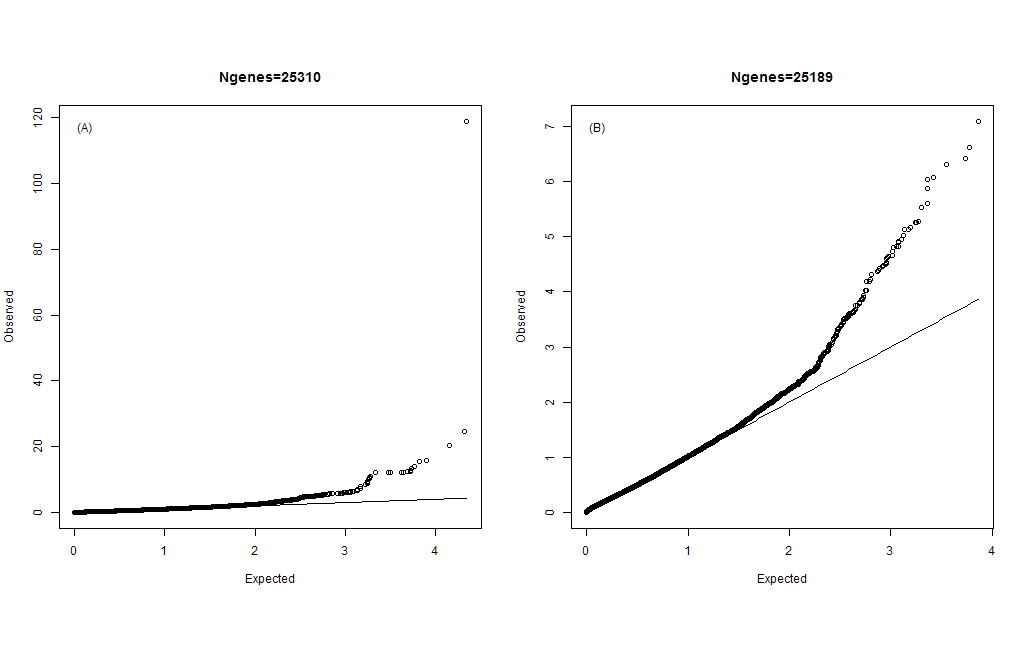

Supplement: Figure S4 — QQ-plot of gene-wide p-values for all genes (A) and excluding previously reported [4]-[8] GWAS significantly associated genes ±0.5Mb (B) in the discovery dataset. Genomic control λ = 1.08 and 1.07 respectively. (TIFF) [file pone.0094661.s015.tif]
