# Supplementary material for: Gene-Wide Analysis Detects Two New Susceptibility Genes for Alzheimer's Disease
Source: PLoS One. 2014 Jun 12;9(6):e94661. doi: 10.1371/journal.pone.0094661 (PMC4055488; doi:10.1371/journal.pone.0094661)
Supplement: Methods S1 — Expression quantitative trait loci (eQTL) and Methylation quantitative trait loci (meQTL) analyses. (DOCX) [file pone.0094661.s016.docx]

**Methods S1.**

# *Expression quantitative trait loci (eQTL) analysis*

We identified alias rsIDs for our significant SNPs at the *TP53INP1* and *IGHV1-67* loci using SNAP[1]. Further proxy SNPs in linkage disequilibrium (r^2^>0.8) were identified in the HapMap 1000 Genomes build (CEU population) with SNAP. SNP rsIDs and aliases were searched for primary SNPs and LD proxies against a collected database of expression SNP (eSNP) results, focusing only on brain tissue datasets. The collected eSNP results met criteria for statistical thresholds for association with gene transcript levels as described in the original papers.

Brain tissue results sources included the following: cortex [2–4], pre-frontal cortex [5–7], parietal lobe [8], frontal cortex [6,9], temporal cortex [4,6,9], hippocampus [6], thalamus [6], pons [9], cerebellum [4,6,8,9], and 3 additional large studies of brain regions including prefrontal cortex, visual cortex and cerebellum in Alzheimer’s disease cases and normal controles [10]. Additional eQTL data was integrated from ScanDB ([www.scandb.org](http://www.scandb.org)). Cerebellum and parietal lobe eQTL data was downloaded from ScanDB and cis-eQTLs were limited to those with P<1.0E-6 and trans-eQTLs with P<5.0E-8.

# *Methylation quantitative trait loci (meQTL) analysis*

Index SNPs and proxies were queried against previously published methylation QTLs (meQTLs). The meQTLs originated from cerebellum [9,11], frontal cortex [9], temporal cortex [9], and pons [9].

Reference List

1. Johnson AD, Handsaker RE, Pulit SL, Nizzari MM, O'Donnell CJ, de Bakker PI (2008) SNAP: a web-based tool for identification and annotation of proxy SNPs using HapMap. Bioinformatics 24: 2938-2939. btn564 [pii];10.1093/bioinformatics/btn564 [doi].

2. Heinzen EL, Ge D, Cronin KD, Maia JM, Shianna KV, Gabriel WN, Welsh-Bohmer KA, Hulette CM, Denny TN, Goldstein DB (2008) Tissue-specific genetic control of splicing: implications for the study of complex traits. PLoS Biol 6: e1. 08-PLBI-RA-1982 [pii] 10.1371/journal.pbio.1000001.

3. Webster JA, Gibbs JR, Clarke J, Ray M, Zhang W, Holmans P, Rohrer K, Zhao A, Marlowe L, Kaleem M, McCorquodale DS, III, Cuello C, Leung D, Bryden L, Nath P, Zismann VL, Joshipura K, Huentelman MJ, Hu-Lince D, Coon KD, Craig DW, Pearson JV, Heward CB, Reiman EM, Stephan D, Hardy J, Myers AJ (2009) Genetic control of human brain transcript expression in Alzheimer disease. Am J Hum Genet 84: 445-458. S0002-9297(09)00108-6 [pii] 10.1016/j.ajhg.2009.03.011.

4. Zou F, Chai HS, Younkin CS, Allen M, Crook J, Pankratz VS, Carrasquillo MM, Rowley CN, Nair AA, Middha S, Maharjan S, Nguyen T, Ma L, Malphrus KG, Palusak R, Lincoln S, Bisceglio G, Georgescu C, Kouri N, Kolbert CP, Jen J, Haines JL, Mayeux R, Pericak-Vance MA, Farrer LA, Schellenberg GD, Petersen RC, Graff-Radford NR, Dickson DW, Younkin SG, Ertekin-Taner N (Brain expression genome-wide association study (eGWAS) identifies human disease-associated variants. PLoS Genet 8: e1002707. 10.1371/journal.pgen.1002707 PGENETICS-D-11-02220 [pii].

5. Colantuoni C, Lipska BK, Ye T, Hyde TM, Tao R, Leek JT, Colantuoni EA, Elkahloun AG, Herman MM, Weinberger DR, Kleinman JE (Temporal dynamics and genetic control of transcription in the human prefrontal cortex. Nature 478: 519-523. nature10524 [pii] 10.1038/nature10524.

6. Kim S, Cho H, Lee D, Webster MJ (2012) Association between SNPs and gene expression in multiple regions of the human brain. Transl Psychiatry 2: e113. tp201242 [pii];10.1038/tp.2012.42 [doi].

7. Liu C, Cheng L, Badner JA, Zhang D, Craig DW, Redman M, Gershon ES (Whole-genome association mapping of gene expression in the human prefrontal cortex. Mol Psychiatry 15: 779-784. mp2009128 [pii] 10.1038/mp.2009.128.

8. Gamazon ER, Badner JA, Cheng L, Zhang C, Zhang D, Cox NJ, Gershon ES, Kelsoe JR, Greenwood TA, Nievergelt CM, Chen C, McKinney R, Shilling PD, Schork NJ, Smith EN, Bloss CS, Nurnberger JI, Edenberg HJ, Foroud T, Koller DL, Scheftner WA, Coryell W, Rice J, Lawson WB, Nwulia EA, Hipolito M, Byerley W, McMahon FJ, Schulze TG, Berrettini WH, Potash JB, Zandi PP, Mahon PB, McInnis MG, Zollner S, Zhang P, Craig DW, Szelinger S, Barrett TB, Liu C (2013) Enrichment of cis-regulatory gene expression SNPs and methylation quantitative trait loci among bipolar disorder susceptibility variants. Mol Psychiatry 18: 340-346. mp2011174 [pii];10.1038/mp.2011.174 [doi].

9. Gibbs JR, van der Brug MP, Hernandez DG, Traynor BJ, Nalls MA, Lai SL, Arepalli S, Dillman A, Rafferty IP, Troncoso J, Johnson R, Zielke HR, Ferrucci L, Longo DL, Cookson MR, Singleton AB (Abundant quantitative trait loci exist for DNA methylation and gene expression in human brain. PLoS Genet 6: e1000952. 10.1371/journal.pgen.1000952.

10. Zhang B, Gaiteri C, Bodea LG, Wang Z, McElwee J, Podtelezhnikov AA, Zhang C, Xie T, Tran L, Dobrin R, Fluder E, Clurman B, Melquist S, Narayanan M, Suver C, Shah H, Mahajan M, Gillis T, Mysore J, MacDonald ME, Lamb JR, Bennett DA, Molony C, Stone DJ, Gudnason V, Myers AJ, Schadt EE, Neumann H, Zhu J, Emilsson V (2013) Integrated systems approach identifies genetic nodes and networks in late-onset Alzheimer's disease. Cell 153: 707-720. S0092-8674(13)00387-5 [pii];10.1016/j.cell.2013.03.030 [doi].

11. Zhang D, Cheng L, Badner JA, Chen C, Chen Q, Luo W, Craig DW, Redman M, Gershon ES, Liu C (2010) Genetic control of individual differences in gene-specific methylation in human brain. Am J Hum Genet 86: 411-419. S0002-9297(10)00087-X [pii];10.1016/j.ajhg.2010.02.005 [doi].
