# Supplementary material for: Gene-Wide Analysis Detects Two New Susceptibility Genes for Alzheimer's Disease
Source: PLoS One. 2014 Jun 12;9(6):e94661. doi: 10.1371/journal.pone.0094661 (PMC4055488; doi:10.1371/journal.pone.0094661)
Supplement: Materials S1 — Full IGAP datasets description. (DOCX) [file pone.0094661.s017.docx]

**GERAD consortium**

Jade Chapman^1^, Alexandra Stretton^1^, Angharad Morgan^1^, Patrick G Kehoe^2^, Christopher Medway^3^, Jenny Lord^3^, James Turton^3^, Nigel M Hooper^4^, Emma Vardy^5^, Jason D Warren^6^, Jonathan M Schott^6^, James Uphill^7^, Natalie Ryan^6^, Martin Rossor^6;7^, Yoav Ben-Shlomo^8^, Daniilidou Makrina^9^, Olymbia Gkatzima^9^, Michelle Lupton^10^, Maria Koutroumani^9^, Despoina Avramidou^9^, Antonia Germanou^9^, Frank Jessen^11^, Ste_Riedel-Heller^12^, Martin Dichgans^13^, Reiner Heun^14^, Heike Kölsch^14^, Britta Schürmann^14^, Christine Herold^15^, André Lacour^15^, Dmitriy Drichel^15^, Per Hoffmann^16^, Johannes Kornhuber^17^, Thomas Feulner^18^, Hendrik van den Bussche^19^, Brian Lawlor^20^, Aoibhinn Lynch^20^, David Mann^21^, David A Smith^22^, Donald Warden^22^, Gordon Wilcock^22^, Isabella Heuser^23^, Jens Wiltfang^24^, Lutz Frölich^25^, Michael Hüll^26^, Kevin Mayo^27^, Gill Livingston^28^, Nicholas J Bass^28^, Hugh Gurling^28^, Andrew McQuillin^28^, Rhian Gwilliam^29^, Panagiotis Deloukas^29^, Ammar Al-Chalabi^30^, Christopher E Shaw^30^, Andrew B Singleton^31^, Rita Guerreiro^31^, Karl-Heinz Jöckel^32^, Norman Klopp^33^, H-Erich Wichmann^33^, Dennis W Dickson^34^, Neill R Graff-Radford^34^, Li Ma^34^, Gina Bisceglio^34^, Elizabeth Fisher^35^, Nick Warner^36^, Stuart Pickering-Brown^37^.

1. Institute of Psychological Medicine and Clinical Neurosciences, MRC Centre for Neuropsychiatric Genetics & Genomics, Cardiff University, UK

2. University of Bristol Institute of Clinical Neurosciences, School of Clinical Sciences, Frenchay Hospital, Bristol, UK

3. Institute of Genetics, Queen’s Medical Centre, University of Nottingham, UK.

4. Institute of Molecular and Cellular Biology, Faculty of Biological Sciences, LIGHT Laboratories, University of Leeds, Leeds, UK.

5. Institute for Ageing and Health, Newcastle University, Biomedical Research Building, Campus for Ageing and Vitality, Newcastle upon Tyne.

6. Dementia Research Center, Department of Neurodegenerative Disease, UCL Institute of Neurology, London, UK.

7. MRC Prion Unit, Department of Neurodegenerative Disease, UCL Institute of Neurology, London, UK.

8. School of Social and Community Medicine, University of Bristol, Bristol, UK

9. Aristotle University of Thessaloniki, Despere 3, Thessaloniki, 54621, Greece

10. King’s College London, Institute of Psychiatry, Department of Neuroscience, De Crespigny Park, Denmark Hill, London

11. Department of Psychiatry and Psychotherapy, University of Bonn, Germany and German Center for Neurodegenerative Diseases (DZNE, Bonn), Bonn, Germany

12. Institute of Social Medicine, Occupational Health and Public Health, University of Leipzig, Leipzig, Germany

13. Institute for Stroke and Dementia Research, Klinikum der Universität München, Munich, Germany and German Center for Neurodegenerative Diseases (DZNE, Munich), Munich, Germany

14. Department of Psychiatry and Psychotherapy, University of Bonn, Germany

15. Deutsches Zentrum für Neurodegenerative Erkrankungen (DZNE, Bonn), Bonn, Germany

16. Division of Medical Genetics, University Hospital and Department of Biomedicine, University of Basel, Basel, Switzerland

17. Department of Psychiatry, University of Erlangen, Nürnberg, Germany

18. Dept. Of Psychaitry, University Hospital, Saarland, Germany

19. Institute of Primary Medical Care, University Medical Center Hamburg-Eppendorf, Germany

20. Mercer’s Institute for Research on Aging, St. James Hospital and Trinity College, Dublin, Ireland

21. Clinical Neuroscience Research Group, Greater Manchester Neurosciences Centre, University of Manchester, Salford, UK

22. Oxford Project to Investigate Memory and Ageing (OPTIMA), University of Oxford, Level 4, John Radclie Hospital, Oxford OX3 9DU, UK

23. Department of Psychiatry, Charité, Berlin, Germany

24. LVR-Hospital Essen, Department of Psychiatry and Psychotherapy, University Duisburg-Essen, Germany

25. Central Institute of Mental Health, Medical Faculty Mannheim, University of Heidelberg, Germany

26. Department of Psychiatry, University of Frankfurt am Main, Frankfurt am Main, Germany

27. Departments of Psychiatry, Neurology and Genetics, Washington University School of Medicine, St Louis, MO 63110, US.

28. Department of Mental Health Sciences, University College London, UK.

29. The Wellcome Trust Sanger Institute, Wellcome Trust Genome Campus, Hinxton, Cambridge, UK

30. MRC Centre for Neurodegeneration Research, Department of Clinical Neuroscience, King’s College London, Institute of Psychiatry, London, SE5 8AF, UK

31. Laboratory of Neurogenetics, National Institute on Aging, National Institutes of Health, Bethesda, MD, 20892, USA.

32. Institute for Medical Informatics, Biometry and Epidemiology, University Hospital of Essen, University Duisburg-Essen, Hufelandstr. 55, D-45147 Essen, Germany

33. Institute of Epidemiology, Helmholtz Zentrum München, German Research Center for Environmental Health, Neuherberg, Germany

34. Department of Neuroscience, Mayo Clinic, Jacksonville, Florida, 32224, USA

35. Department of Neurodegenerative Disease, UCL Institute of Neurology, London, UK

36. Somerset Partnership NHS Trust, Somerset, UK

37. Institute of Brain, Behaviour and Mental Health, Faculty of Human and Medical Sciences, University of Manchester, Manchester UK

**EADI consortium**

Annick Alpérovitch^1^, Anne Boland^2^, Marc Delépine^2^, Bruno Dubois^3^, Emmanuelle Duron^4^, Jacques Epelbaum^5^, Caroline Van Cauwenberghe^6;7^, Sebastiaan Engelborghs^7;8^, Rik Vandenberghe^9;10^, Peter P. De Deyn^7;8^, Raffaele Ferri^11^, Carmelo Romano^11^, Carlo Caltagirone^12^, Maria Donata Orfei^12^, Antonio Ciaramella^12^, Elio Scarpini^13;14^, Chiara Fenoglio^13;14^, Gloria Tognoni^15^, Silvia Bagnoli^16;17^, Laura Bracco^16;17^, Valentina Bessi^16;17^, Roberta Cecchetti^18;19^, Patrizia Bastiani1^8;19^, Alessio Squassina^20^, Davide Seripa^21^, Isabel Sastre^23;24,25^, Rafael Blesa^26;27^, Daniel Alcolea^26;27^, Marc Suárez-Calvet^26;27^, Pascual Sánchez-Juan^28^, Carmen Muñoz Fernandez^29^, Yolanda Aladro Benito^29^, Håkan Thonberg^30^, Charlotte Forsell^31^, Lena Lilius^31^, Anne Kinhult-Ståhlbom^30^, Vilmantas Giedraitis^32^, Lena Kilander^32^, Rose Marie Brundin^32^, Letizia Concari^33;34^, Seppo Helisalmi^35;36^, Anne Maria Koivisto^35;36^, Annakaisa Haapasalo^35;36^, Vincenza Frisardi^37^, Jurg Ott^38^.

1. Inserm U708, Victor Segalen University, F-33076, Bordeaux, France

2. Centre National de Genotypage, Institut Genomique, Commissariat à l’énergie Atomique, Evry, France

3. IM2A - CRicm-UMRS975, AP-HP, Hôpital de la Pitié-Salpêtrière, Paris, France

4. CMRR Paris Sud Ile-de-France, service de gériatrie, Hôpital Broca, 54-56 rue Pascal, 75013 Paris

5. UMR 894 Inserm, Psychiatry and Neurosciences Center, Paris, France

6. Neurodegenerative Brain Diseases Group, Department of Molecular Genetics, VIB, Antwerp, Belgium

7. Institute Born-Bunge, University of Antwerp, Antwerp, Belgium

8. Department of Neurology and Memory Clinic, Hospital Network Antwerp, Antwerp, Belgium

9. Laboratory for Cognitive Neurology, Department of Neurology, University of Leuven, Leuven, Belgium

10. Department of Neurology and Memory Clinic, University Hospitals Leuven Gasthuisberg, Leuven, Belgium

11. IRCCS Associazione Oasi Maria SS, Troina, Italy

12. Clinical and Behavioral Neurology, Fondazione Santa Lucia, Roma ,Italy

13. University of Milan, Milan, Italy

14. Fondazione Cà Granda, IRCCS Ospedale Policlinico, Milan, Italy

15. Neurological Clinic, University of Pisa, Italy

16. Department of Neurological and Psychiatric Sciences, University of Florence, Florence, Italy

17. Centro di Ricerca, Trasferimento e Alta Formazione DENOTHE, University of Florence, Florence, Italy

18. Section of Gerontology and Geriatrics, Department of Clinical and Experimental Medicine Perugia, Italy

19. University of Perugia, Perugia, Italy

20. Section of Neuroscience and Clinical Pharmacology, Department of Biomedical Science, University of Cagliari, Cagliari, Italy

21. Gerontology and Geriatrics Research Laboratory, I.R.C.C.S. Casa Sollievo della So_erenza, San Giovanni Rotondo (FG), Italy

22. Neurology Service; Hospital Universitario La Paz (UAM), Madrid, Spain

23. Centro de Investigación Biomédica en Red sobre Enfermedades Neurodegenerativas (CIBERNED), Madrid, Spain

24. Instituto de Investigación Sanitaria «Hospital la Paz» (IdIPaz), Madrid, Spain

25. Centro de Biología Molecular Severo Ochoa (CSIC-UAM), Madrid, Spain

26. Neurology Department. IIB Sant Pau. Sant Pau Hospital. Universitat Autònoma de Barcelona. Barcelona, Spain.

27. Center for Networker Biomedical Research in Neurodegenerative Diseases (CIBERNED), Barcelona, Spain.

28. Neurology Service and CIBERNED, «Marqués de Valdecilla» University Hospital (University of Cantabria and IFIMAV), Santander, Spain

29. Department of Neurology, Hospital Universitario Dr. Negrin, Las Palmas de Gran Canaria, Spain

30. Dept Geriatric Medicine, Genetics Unit, Karolinska University Hospital Huddinge, S-14186 Stockholm, Sweden

31. Karolinska Institutet, Dept Neurobiology, Care Sciences and Society, KIADRC, Novum floor 5, S14186 Stockholm, Sweden

32. Dept. of Public Health/Geriatrics, Uppsala University, Uppsala, Sweden

33. Department of Neuroscience-University of Parma-Italy, Parma, Italy

34. Center for Cognitive Disorders AUSL, Parma, Italy

35. Institute of Clinical Medicine - Neurology, University of Eastern Finland, FIN-70211, Kuopio, Finland

36. Department of Neurology, Kuopio University Hospital, FIN-70211 Kuopio, Finland

37. Department of Neurosciences and Sensory Organs, Aldo Moro University of Bari, Bari, Italy

38. Laboratory of Statistical Genetics, The Rockfeller University, 1230 York Avenue, New York, NY, 10065, USA

**ADGC consortium**

Regina M Carney^1^, Deborah C Mash^2^, Marilyn S Albert^3^, Roger L Albin^4;5^, Liana G Apostolova^6^, Steven E Arnold^7^, Michael M Barmada^8^, Lisa L Barnes^9;10^, Thomas G Beach^11^, Eileen H Bigio^12^, Thomas D Bird^13^, Bradley F Boeve^14^, James D Bowen^15^, Adam Boxer^16^, James R Burk^17^, Nigel J Cairns^18^, Chuanhai Cao^19^, Chris S Carlson^20^, Steven L Carroll^21^, Lori B Chibnik^22;23^, Helena C Chui^24^, David G Clark^25^, Jason Corneveaux^26^, David G Cribbs^27^, Charles DeCarli^28^, Steven T DeKosky^29^, F Yesim Demirci^8^, Malcolm Dick^30^, Dennis W Dickson^31^, Ranjan Duara^32^, Nilufer Ertekin-Taner^31;33^, Kenneth B Fallon^21^, Martin R Farlow^34^, Steven Ferris^35^, Matthew P Frosch^36^, Douglas R Galasko^37^, Mary Ganguli^38^, Marla Gearing^39;40^, Daniel H Geschwind^41^, Bernardino Ghetti^42^, Sid Gilman^4^, Jonathan D Glass^43^, John H Growdon^44^, Ronald L Hamilton^45^, Lindy E Harrell^46^, Elizabeth Head^47^, Lawrence S Honig^48^, Christine M Hulette^49^, Bradley T Hyman^44^, Gail P Jarvik^50;51^, Gregory A Jicha^52^, Lee-Way Jin^53^, Anna Karydas^16^, John SK Kauwe^54^, Jeffrey A Kaye^55;56^, Ronald Kim^57^, Edward H Koo^37^, Neil W Kowall^58;59^, Joel H Kramer^60^, Patricia Kramer^55;61^, Frank M LaFerla^62^, James J Lah^43^, James B Leverenz^63^, Allan I Levey^43^, Ge Li^64^, Andrew P Lieberman^65^, Constantine G Lyketsos^66^, Wendy J Mack^67^, Daniel C Marson^46^, Frank Martiniuk^68^, Eliezer Masliah^37;69^, Wayne C McCormick^70^, Susan M McCurry^71^, Andrew N McDavid^20^, Ann C McKee^58;59^, Marsel Mesulam^72^, Bruce L Miller^16^, Carol A Miller^73^, Joshua W Miller^74^, John C Morris^18;75^, the Alzheimer’s Disease Neuroimaging Initiative*, Jill R Murrell^42;76^, John M Olichney^77^, Vernon S Pankratz^78^, Joseph E Parisi^79;80^, Elaine Peskind^64^, Ronald C Petersen^81^, Aimee Pierce^27^, Wayne W Poon^30^, Huntington Potter^82^, Joseph F Quinn^55^, Ashok Raj^82^, Murray Raskind^64^, Eric M. Reiman^26;83;84^, Barry Reisberg^35;85^, John M Ringman^6^, Erik D Roberson^46^, Howard J Rosen^16^, Roger N Rosenberg^86^, Mary Sano^87^, Andrew J Saykin^42;88^, Julie A Schneider^9;89^, Lon S Schneider^6;90^, William W Seeley^16^, Amanda G Smith^82^, Joshua A Sonnen^63^, Salvatore Spina^42^, Robert A Stern^58^, Rudolph E Tanzi^44^, John Q Trojanowski^91^, Juan C Troncoso^92^, Vivianna M Van Deerlin91, Linda J Van Eldik^93^, Harry V Vinters^6;94^, Jean Paul Vonsattel^95^, Sandra Weintraub^72^, Kathleen A Welsh-Bohmer^96;97^, Jennifer Williamson^48^, Randall L Woltjer^98^, Chang-En Yu^70^, Robert Barber^99^, Robert C. Green^22^, John Gilbert^6,90^

1. The John P. Hussman Institute for Human Genomics, University of Miami, Miami, FL, 33124, USA

2. Department of Neurology, University of Miami, Miami, FL, 33124, USA

3. Department of Neurology, Johns Hopkins University, Baltimore, MD, 21218, USA

4. Department of Neurology, University of Michigan, Ann Arbor, MI, 48109, USA

5. Geriatric Research, Education and Clinical Center (GRECC), VA Ann Arbor Healthcare System (VAAAHS), Ann Arbor, MI, 48109, USA

6. Department of Neurology, University of California Los Angeles, Los Angeles, CA, 94607, USA

7. Department of Psychiatry, University of Pennsylvania Perelman School of Medicine, Philadelphia, PA, 19104, USA

8. Department of Human Genetics, University of Pittsburgh, Pittsburgh, PA, 15213, USA

9. Department of Neurological Sciences, Rush University Medical Center, Chicago, IL, 60612, USA

10. Department of Behavioral Sciences, Rush University Medical Center, Chicago, IL, 60612, USA

11. Civil Laboratory for Neuropathology, Banner Sun Health Research Institute, Phoenix, AZ, 85351, USA

12. Department of Pathology, Northwestern University, Chicago, IL, 60208, USA

13. Department of Neurology, University of Washington, Seattle, WA, 98195, USA

14. Department of Neurology, Mayo Clinic, Rochester, MN, 55902, USA

15. Swedish Medical Center, Seattle, WA, 98195, USA

16. Department of Neurology, University of California San Francisco, San Francisco, CA, 94122, USA

17. Department of Medicine, Duke University, Durham, NC, 27710, USA

18. Department of Pathology and Immunology, Washington University, St. Louis, MO, 63130, USA

19. USF Health Byrd Alzheimer’s Institute, University of South Florida, Tampa, CA, 33620, USA

20. Fred Hutchinson Cancer Research Center, Seattle, WA, 98195, USA

21. Department of Pathology, University of Alabama at Birmingham, Birmingham, AL, 35294, USA

22. Program in Translational NeuroPsychiatric Genomics, Institute for the Neurosciences, Department of Neurology & Psychiatry, Brigham and Women’s Hospital, Harvard Medical School, Boston, MA, 02215, USA

23. Program in Medical and Population Genetics, Broad Institute, Boston, MA, 02215, USA

24. Department of Neurology, University of Southern California, Los Angeles, CA, 94607, USA

25. Department of Neurology, University of Alabama at Birmingham, Birmingham, AL, 35294, USA

26. Neurogenomics Division, Translational Genomics Research Institute, Phoenix, AZ, 85004, USA

27. Department of Neurology, University of California Irvine, Irvine, CA, 92617, USA

28. Department of Neurology, University of California Davis, Sacramento, CA, 95616, USA

29. University of Virginia School of Medicine, Charlottesville, VA, 22903, USA

30. Institute for Memory Impairments and Neurological Disorders, University of California Irvine, Irvine, CA, 92617, USA

31. Department of Neuroscience, Mayo Clinic, Jacksonville, FL, 55902, USA

32. Wien Center for Alzheimer’s Disease and Memory Disorders, Mount Sinai Medical Center, Miami Beach, FL, 10029, USA

33. Department of Neurology, Mayo Clinic, Jacksonville, FL, 55902, USA

34. Department of Neurology, Indiana University, Indianapolis, IN, 46202, USA

35. Department of Psychiatry, New York University, New York, NY, 10027, USA

36. C.S. Kubik Laboratory for Neuropathology, Massachusetts General Hospital, Charlestown, MA, 02114, USA

37. Department of Neurosciences, University of California San Diego, La Jolla, CA, 92093, USA

38. Department of Psychiatry, University of Pittsburgh, Pittsburgh, PA, 15213, USA

39. Department of Pathology and Laboratory Medicine, Emory University, Atlanta, GA, 30329, USA

40. Emory Alzheimer’s Disease Center, Emory University, Atlanta, GA, 30329, USA

41. Neurogenetics Program, University of California Los Angeles, Los Angeles, CA, 94607, USA

42. Department of Pathology and Laboratory Medicine, Indiana University, Indianapolis, IN, 46202, USA

43. Department of Neurology, Emory University, Atlanta, GA, 30329, USA

44. Department of Neurology, Massachusetts General Hospital/Harvard Medical School, Boston, MA, 02215, USA

45. Department of Pathology (Neuropathology), University of Pittsburgh, Pittsburgh, PA, 15213, USA

46. Department of Neurology, University of Alabama at Birmingham, Birmingham, AL, 35294, USA

47. Sanders-Brown Center on Aging, Department of Molecular and Biomedical Pharmacology, University of Kentucky, Lexington, KY, 40536, USA

48. Taub Institute on Alzheimer’s Disease and the Aging Brain, Department of Neurology, Columbia University, New York, NY, 10027, USA

49. Department of Pathology, Duke University, Durham, NC, 27710, USA

50. Department of Genome Sciences, University of Washington, Seattle, WA, 98195, USA

51. Department of Medicine (Medical Genetics), University of Washington, Seattle, WA, 98195, USA

52. Sanders-Brown Center on Aging, Department Neurology, University of Kentucky, Lexington, KY, 40536, USA

53. Department of Pathology and Laboratory Medicine, University of California Davis, Sacramento, CA, 95616, USA

54. Department of Biology, Brigham Young University, Provo, UT 02215, USA

55. Department of Neurology, Oregon Health & Science University, Portland, OR, 97239, USA

56. Department of Neurology, Portland Veterans Affairs Medical Center, Portland, OR, 97239, USA

57. Department of Pathology and Laboratory Medicine, University of California Irvine, Irvine, CA, 92617, USA

58. Department of Neurology, Boston University School of Medicine, Boston, MA, 02215, USA

59. Department of Pathology, Boston University School of Medicine, Boston, MA, 02215, USA

60. Department of Neuropsychology, University of California San Francisco, San Francisco, CA, 94122, USA

61. Department of Molecular & Medical Genetics, Oregon Health & Science University, Portland, OR, 97239, USA

62. Department of Neurobiology and Behavior, University of California Irvine, Irvine, CA, 92617, USA

63. Department of Pathology, University of Washington, Seattle, WA, 98195, USA

64. Department of Psychiatry and Behavioral Sciences, University of Washington, Seattle, WA, 98195, USA

65. Department of Pathology, University of Michigan, Ann Arbor, MI, 48109, USA

66. Department of Psychiatry, Johns Hopkins University, Baltimore, MD, 21218, USA

67. Department of Preventive Medicine, University of Southern California, Los Angeles, CA, 94607, USA

68. Department of Medicine - Pulmonary, New York University, New York, NY, 10027, USA

69. Department of Pathology, University of California San Diego, La Jolla, CA, 92093, USA

70. Department of Medicine, University of Washington, Seattle, WA, 98195, USA

71. School of Nursing Northwest Research Group on Aging, University of Washington, Seattle, WA, 98195, USA

72. Cognitive Neurology and Alzheimer’s Disease Center, Northwestern University, Chicago, IL, 60208, USA

73. Department of Pathology, University of Southern California, Los Angeles, CA, 94607, USA

74. Department of Pathology and Laboratory Medicine, University of California Davis, Sacramento, CA, 95616, USA

75. Department of Neurology, Washington University, St. Louis, MO, 98101, USA

76. Department of Medical and Molecular Genetics, Indiana University, Indianapolis, IN, 46202, USA

77. Department of Neurology, University of California Davis, Sacramento, CA, 95616, USA

78. Department of Biostatistics, Mayo Clinic, Rochester, MN, 55902, USA

79. Department of Anatomic Pathology, Mayo Clinic, Mayo Clinic, Rochester, MN, 55902, USA

80. Department of Laboratory Medicine and Pathology, Mayo Clinic, Rochester, MN, 55902, USA

81. Department of Neurology, Mayo Clinic, Rochester, MN, 55902, USA

82. USF Health Byrd Alzheimer’s Institute, University of South Florida, Tampa, FL, 33613, USA

83. Arizona Alzheimer’s Consortium, Department of Psychiatry, University of Arizona, Phoenix, AZ, 85004, USA

84. Banner Alzheimer’s Institute, Phoenix, AZ, 85004, USA

85. Alzheimer’s Disease Center, New York University, New York, NY, 10027, USA

86. Department of Neurology, University of Texas Southwestern, Dallas, TX, 75390, USA

87. Department of Psychiatry, Mount Sinai School of Medicine, New York, NY, 10027, USA

88. Department of Radiology and Imaging Sciences, Indiana University, Indianapolis, IN, 46202, USA

89. Department of Pathology (Neuropathology), Rush University Medical Center, Chicago, IL, 60208, USA

90. Department of Psychiatry, University of Southern California, Los Angeles, CA, 94607, USA

91. Department of Pathology and Laboratory Medicine, University of Pennsylvania, Perelman School of Medicine, Philadelphia, PA, 19104, USA

92. Department of Pathology, Johns Hopkins University, Baltimore, MD, 21218, USA

93. Sanders-Brown Center on Aging, Department of Anatomy and Neurobiology, University of Kentucky, Lexington, KY, 40536, USA

94. Department of Pathology & Laboratory Medicine, University of California Los Angeles, Los Angeles, CA, 94607, USA

95. Taub Institute on Alzheimer’s Disease and the Aging Brain, Department of Pathology, Columbia University, New York, NY, 10027, USA

96. Department of Medicine, Duke University, Durham, NC, 27710, USA

97. Department of Psychiatry & Behavioral Sciences, Duke University, Durham, NC, 27710, USA

98. Department of Pathology, Oregon Health & Science University, Portland, OR, 97239, USA

99. Department of Pharmacology and Neuroscience, University of North Texas Health Science Center, FortWorth, TX, 76102, USA

**CHARGE consortium**

Rhoda Au^1^, Philip A Wolf^1^, Alexa Beiser^2^, Stephanie Debette^3;4^, Qiong Yang^2^, Galit Weinstein^1^, Jing Wang^2^, Andre G Uiterlinden^5;6;7^, Hieab H.H. Adams^6^, Fernando Rivadeneira^5^, Peter J Koudstaal^8^, William T Longstreth Jr^9;10;11^, James T Becker^12^, Lewis H Kuller^13^, Thomas Lumley^14^, Kenneth Rice^15^, Melissa Garcia^16^, Thor Aspelund^17^, Josef JM Marksteiner^18^, Peter Dal-Bianco^19^, Anna Maria Töglhofer^20^, Paul Freudenberger^20^, Gerhard Ransmayr^21^, Thomas Benke^22^, Anna M Toeglhofer^20^, Jan Bressler^23^, Monique MB Breteler^24^, Myriam Fornage^25^, Isabel Hernández^26^, Maitee Rosende Roca^26^, Ana Mauleón^26^, Montserrat Alegret^26^, Reposo Ramírez-Lorca^27^, Antonio González-Perez^27^.

1. Department of Neurology, Boston University School of Medicine, Boston, MA 2118, USA

2. Department of Biostatistics, Boston University School of Public Health, Boston, MA 2118, USA

3. Department of Neurology, Boston University School of Medicine, Boston, MA 2118, USA

4. UMR744 Inserm, Lille, France

5. Department of Internal Medicine, Erasmus MC University Medical Center, Rotterdam, 3000 CA, The Netherlands

6. Department of Epidemiology, Erasmus University Medical Center, PO Box 2040, 3000 CA Rotterdam, The Netherlands

7. Netherlands Consortium for Healthy Aging (NCHA), Leiden, The Netherlands

8. Department of Neurology, Erasmus MC University Medical Center, Rotterdam 3000 CA, The Netherlands

9. Cardiovascular Health Research Unit, Department of Medicine, University ofWashington, Seattle,WA 98101, USA

10. Department of Epidemiology, University of Washington, Seattle, WA 98101, USA

11. Group Health Research Institute, Group Health Cooperative, Seattle, WA 98101, USA

12. The Departments of Neurology and Psychiatry, University of Pittsburgh School of Medicine, Pittsburgh, PA 15213 USA

13. Departments of Medicine and Epidemiology, University of Pittsburgh School of Medicine, Pittsburgh, PA 15213, USA

14. Department of Statistics, University of Auckland, Auckland, New Zealand.

15. Department of Biostatistics, University of Washington, School of Medicine, Seattle, WA 98101, USA

16. Laboratory of Epidemiology, Demography and Biometry, National Institute on Aging, Bethesda, MD, 20892, USA

17. The Icelandic Heart Association, Landspitali-University Hospital, Reykjavik, Iceland

18. Department of Psychiatry and Psychotherapy, General Hospital, Hall, 6060, Austria

19. Department of Neurology, Medical University of Vienna, Vienna, 1090, Austria

20. Institute for Molecular Biology and Biochemistry, Medical University of Graz, Graz, 8010, Austria

21. Department of Neurology and Psychiatry,General Hospital Linz, Austria, Linz, 4020, Austria

22. Clinic of Neurology, Medical University Innsbruck, Innsbruck, 6020, Austria

23. School of Public Health, University of Texas Health Sciences Center at Houston, Houston, TX 77030, USA

24. German Center for Neurodegenerative diseases (DZNE), Bonn, 53175, Germany

25. Institute of Molecular Medicine and School of Public Health Division of Epidemiology Human Genetics and Environmental Sciences, University of Texas Health Sciences Center at Houston, Houston, TX 77030, USA

26. Memory Clinic of Fundació ACE. Institut Català de Neurociències Aplicades, Barcelona, 8029, Spain

27. Departamento de Genómica Estructural . Neocodex, Sevilla, 8029, Spain

**UK Brain Expression consortium**

John Hardy^1^, Adaikalavan Ramasamy^2^, Mina Ryten^1,2^, Colin Smith^3^, Daniah Trabzuni^1,4^, Robert Walker^3^, Michael E. Weale^2^

1. Reta Lilla Weston Laboratories, Department of Molecular Neuroscience, UCL Institute of Neurology, Queen Square, London WC1N 3BG, UK

2. Department of Medical and Molecular Genetics, King’s College London, Guy’s Hospital, 8th Floor, Tower Wing, London SE1 9RT, UK

3. MRC Sudden Death Brain Bank Project, Department of Neuropathology, University of Edinburgh, Wilkie Building, Teviot Place, Edinburgh EH8 9AG, UK

4. Department of Genetics, King Faisal Specialist Hospital and Research Centre, PO Box 3354, Riyadh 11211, Saudi Arabia
