# Supplementary material for: Gene-Wide Analysis Detects Two New Susceptibility Genes for Alzheimer's Disease
Source: PLoS One. 2014 Jun 12;9(6):e94661. doi: 10.1371/journal.pone.0094661 (PMC4055488; doi:10.1371/journal.pone.0094661)
Supplement: Materials S2 — List of IGAP consortium members. (DOC) [file pone.0094661.s018.doc]

**Full I-GAP datasets description**

Written informed consent was obtained from study participants or, for those with substantial cognitive impairment, from a caregiver, legal guardian, or other proxy, and the study protocols for all populations were reviewed and approved by the appropriate Institutional review boards.

**Alzheimer’s Disease Genetics Consortium (ADGC)**

The ADGC dataset comprises subjects from the Adult Changes in Thought (ACT)/ Electronic Medical Recordsand Genetics (eMERGE) study, the National Institute on Aging (NIA) Alzheimer Disease Centers (ADCs), the Alzheimer Disease Neuroimaging Initiative (ADNI) Study*, the Multi-Site Collaborative Study for Genotype-Phenotype Associations in Alzheimers Disease (GenADA) Study, the University of Miami/Vanderbilt University/Mt. Sinai School of Medicine (UM/VU/MSSM), the MIRAGE Study, Oregon Health and Science University (OHSU), the NCRAD/NIA-LOAD Study, the Translational Genomics Research Institute series 2 (TGEN2) dataset, subjects from the Mayo Clinic, the Rush University Religious Orders Study/Memory and Aging Project (ROSMAP), the University of Pittsburgh (UP), and Washington University (WU). Detailed descriptions of the ascertainment and evaluation of subjects in the ADC, ADNI, UM/VU/MSSM, MIRAGE, and NCRAD/NIA-LOAD cohorts have been provided elsewhere [1]; brief descriptions included here note any di_erences between data used in this study and data used in the previously published ADGC study [1]. We restricted analyses to individuals of European ancestry because there were an insu_cient number of subjects from other ethnic groups to obtain meaningful results. All subjects were recruited under protocols approved by the appropriate Institutional Review Boards.

*Data used in preparation of this article were obtained from the Alzheimer’s Disease Neuroimaging Initiative (ADNI) database (adni.loni.usc.edu). As such, the investigators within the ADNI contributed to the design and implementation of ADNI and/or provided data but did not participate in analysis or writing of this report. A complete listing of ADNI investigators can be found at:http://adni.loni.usc.edu/wp-content/uploads/how_to_apply/ADNI_Acknowledgement_List.pdf

**The NIA ADC Samples (ADC):** The NIA ADC cohort included subjects ascertained and evaluated by the clinical and neuropathology cores of the 29 NIA-funded ADCs. Data collection is coordinated by the National Alzheimer’s Coordinating Center (NACC). NACC coordinates collection of phenotype data from the 29 ADCs, cleans all data, coordinates implementation of definitions of AD cases and controls, and coordinates collection of samples. The ADC cohort consists of 2,288 autopsy-confirmed and 913 clinically-confirmed AD cases, and 519 cognitively normal elders (CNEs) with complete neuropathology data who were older than 60 years at age of death, and 744 living CNEs evaluated using the Uniform dataset (UDS) protocol [2, 3] who were documented to not have mild cognitive impairment (MCI) and were between 60 and 100 years of age at assessment. Based on the data collected by NACC, the ADGC Neuropathology Core Leaders Subcommittee derived inclusion and exclusion criteria for AD and control samples. All autopsied subjects were age _ 60 years at death. AD cases were demented according to DSM-IV criteria or Clinical Dementia Rating (CDR) ≥ 1.

Neuropathologic stratification of cases followed NIA/Reagan criteria explicitly, or used a similar approach when NIA/Reagan criteria [4] were coded as not done, missing, or unknown. Cases were intermediate or high likelihood by NIA/Reagan criteria with moderate to frequent amyloid plaques [5] and neurofibrillary tangle (NFT) Braak stage of III-VI [6, 7]. Persons with Down’s syndrome, non-AD tauopathies and synucleinopathies were excluded. All autopsied controls had a clinical evaluation within two years of death. Controls did not meet DSM-IV criteria for dementia, did not have a diagnosis of mild cognitive impairment (MCI), and had a CDR of 0, if performed. Controls were did not meet or were low-likelihood AD by NIA/Reagan criteria, had sparse or no amyloid plaques, and a Braak NFT stage of 0 – II.

ADCs sent frozen tissue from autopsied subjects and DNA samples from some autopsied subjects and from living subjects to the ADCs to the National Cell Repository for Alzheimer’s Disease (NCRAD). DNA was prepared by NCRAD for genotyping and sent to the genotyping site at Children’s Hospital of Philadelphia. ADC samples were genotyped and analyzed in separate batches.

ADC1 and ADC2 contributed 2,304 AD cases (1,761 autopsy-confirmed; 543 clinically-confirmed) and 675 CNEs (515 autopsy-confirmed; 160 clinically-confirmed), of which 1,595 autopsied-confirmed AD cases and 132 CNEs were analyzed in our previous study [1]. The ADC3 dataset contains 897 clinically-identified living cases (527 with autopsy-confirmation) and 588 CNEs (4 with autopsy-confirmation) who were genotyped between July and August 2010.

**Oregon Health and Science University (OHSU):** The OHSU dataset includes 132 autopsy-confirmed AD cases and 153 deceased controls that were evaluated for dementia within 12 months prior to death (age at death >65 years), which are a subset of the 193 cases and 451 controls examined in our previous study [1] meeting more stringent QC criteria in this study. Subjects were recruited from aging research cohorts at 10 NIA-funded ADCs, and did not overlap other samples assembled by the ADGC. A more extensive description of control samples can be found elsewhere [8].

**The ADNI Study (ADNI):** ADNI is a longitudinal, multi-site observational study including AD, mild cognitive impairment (MCI), and elderly individuals with normal cognition assessing clinical and cognitive measures, MRI and PET scans (FDG and 11C PIB) and blood and CNS biomarkers. For this study, ADNI contributed data on 268 AD cases with MRI confirmation of AD diagnosis and 173 healthy controls with AD-free status confirmed as of most recent follow-up. AD subjects were between the ages of 55–90, had an MMSE score of 20–26 inclusive, met NINCDS/ADRDA criteria for probable AD [9], and had an MRI consistent with the diagnosis of AD. Control subjects had MMSE scores between 28 and 30 and a Clinical Dementia Rating of 0 without symptoms of depression, MCI or other dementia and no current use of psychoactive medications. According to the ADNI protocol, subjects were ascertained at regular intervals over 3 years, but for the purpose of our analysis we only used the final ascertainment status to classify case-control status. Additional details of the study design are available elsewhere [1, 10, 11].

Data used in the preparation of this article were obtained from ADNI database ([http://adni.loni.ucla.edu](http://adni.loni.ucla.edu/)). The ADNI was launched in 2003 by the National Institute on Aging (NIA), the National Institute of Biomedical Imaging and Bioengineering (NIBIB), the Food and Drug Administration (FDA), private pharmaceutical companies and non-profit organizations, as a $60 million, 5-year public-private partnership. The primary goal of ADNI has been to test whether serial magnetic resonance imaging (MRI), positron emission tomography (PET), other biological markers, and clinical and neuropsychological assessment can be combined to measure the progression of mild cognitive impairment (MCI) and early Alzheimer’s disease (AD). Determination of sensitive and specific markers of very early AD progression is intended to aid researchers and clinicians to develop new treatments and monitor their effectiveness, as well as lessen the time and cost of clinical trials. The Principal Investigator of this initiative is MichaelW.Weiner, MD, VA Medical Center and University of California – San Francisco. ADNI is the result of efforts of many co-investigators from a broad range of academic institutions and private corporations, and subjects have been recruited from over 50 sites across the U.S. and Canada. The initial goal of ADNI was to recruit 800 subjects but ADNI has been followed by ADNI-GO and ADNI-2. To date these three protocols have recruited over 1500 adults, ages 55 to 90, to participate in the research, consisting of cognitively normal older individuals, people with early or late MCI, and people with early AD. The follow up duration of each group is specified in the protocols for ADNI-1, ADNI-2 and ADNI-GO. Subjects originally recruited for ADNI-1 and ADNI-GO had the option to be followed in ADNI-2. For up-to-date information, see www.adni-info.org.

**The MIRAGE Study (MIRAGE):** The MIRAGE study is a family-based genetic epidemiological study of AD that enrolled AD cases and unaffected sibling controls at 17 clinical centers in the United States, Canada, Germany, and Greece (details elsewhere [12]), and contributed 1,262 subjects (509 AD cases and 753 CNEs), a subset of the 559 cases and 788 controls that were incorporated into our prior study [1] which met more stringent QC criteria for this study. Briefly, families were ascertained through a proband meeting the NINCDS-ADRDA criteria for definite or probable AD. Unaffected sibling controls were verified as cognitively healthy based on a Modified Telephone Interview of Cognitive Status score ≥ 86 [13].

**The NCRAD/NIA-LOAD Family Study (NCRAD/NIA-LOAD):** The NCRAD/NIA-LOAD Family Study [14] recruited families with two or more a_ected siblings with LOAD and unrelated, CNEs similar in age and ethnic background. A total of 1,819 cases and 1,969 CNEs from 1,802 families were recruited through the NCRAD/NIALOAD study, NCRAD, and the University of Kentucky and included for analysis, of which a subset of 985 cases and 881 controls were used in the previous study [1]. One case per family was selected after determining the individual with the strictest diagnosis (definite > probable > possible LOAD). If there were multiple individuals with the strictest diagnosis, then the individual with the earliest age of onset was selected. The controls included only those samples that were neurologically evaluated to be normal and were not related to a study participant.

**University of Miami/Vanderbilt University/Mt. Sinai School of Medicine (UM/VU/MSSM):**

The UM/VU/MSSM dataset contains 1,186 cases and 1,135 CNEs (new and previously published) [15, 16, 17, 18] ascertained at the University of Miami, Vanderbilt University and Mt. Sinai School of Medicine, including 409 autopsy-confirmed cases and 136 controls, primarily from the Mt. Sinai School of Medicine [19]. An additional 16 cases were included and 34 controls excluded from the data analyzed in the prior study [1]. Each affected individual met NINCDS-ADRDA criteria for probably or definite AD with age at onset greater than 60 years as determined from specific probe questions within the clinical history provided by a reliable family informant or from documentation of significant cognitive impairment in the medical record. Cognitively healthy controls were unrelated individuals from the same catchment areas and frequency matched by age and gender, and had a documented MMSE or 3MS score in the normal range. Cases and controls had similar demographics: both had ages-at-onset/ages-at-exam of 74 (± 8 standard deviations), and cases were 63% female, and controls were 61% female.

**The ACT/eMERGE Studies (ACT):** The ACT cohort is an urban and suburban elderly population from a stable HMO that includes 2,581 cognitively intact subjects age ≥ 65 who were enrolled between 1994 and 1998 [20, 21]. An additional 811 subjects were enrolled in 2000-2002 using the same methods except oversampling clinics with more minorities. More recently, a Continuous Enrollment strategy was initiated in which new subjects are contacted, screened and enrolled to keep 2000 active at-risk person-years accruing in each calendar year. This resulted in an enrollment of 4,146 participants as of May 2009. All clinical data are reviewed at a consensus conference. Dementia onset is assigned half way between the prior biennial and the exam that diagnosed dementia. Enrollment for eMERGE Study began in 2007. A waiver of consent was obtained from the IRB to enroll deceased ACT participants. In total, ACT/eMERGE contributed data on 566 individuals with probable or possible AD (70 with autopsy-confirmation) and on 1,696 CNEs (155 with autopsy-confirmation) who were included in analyses.

**The GenADA Study:** Data from the GenADA cohort that were analyzed included 669 AD cases and 713 CNEs ascertained from nine memory referral clinics in Canada between 2002 and 2005. Patients and CNEs were of Caucasian ancestry from Northern Europe. All patients with AD satisfied NINCDS-ADRDA and DSM-IV criteria for probable AD with Global Deterioration Scale scores of 3-7. CNEs had MMSE test scores higher than 25 (mean 29.2 ± 1.1), a Mattis Dementia Rating Scale score of ≥ 136, a Clock Test without error, and no impairments on seven instrumental activities of daily living questions from the Duke Older American Resources and Services Procedures test. Data were collected under an academic-industrial grant from Glaxo-Smith-Kline, Canada by Principal Investigator P. St George-Hyslop. Detailed characteristics of this cohort have been described previously [22].

**The TGEN2 Study:** Among the TGEN2 data analyzed were 864 clinically- and neuropathologically-characterized brain donors, and 493 CNEs without dementia or significant AD pathology. Of these cases and CNEs, 667 were genotyped as a part of the TGEN1 series [23]. Samples were obtained from twenty-one di_erent National Institute on Aging-supported AD Center brain banks and from the Miami Brain Bank as previously described [23, 24, 25]. Additional individual samples from other brain banks in the United States, United Kingdom, and the Netherlands were also obtained in the same manner. The criteria for inclusion were as follows: self-defined ethnicity of European descent, neuropathologically confirmed AD or neuropathology present at levels consistent with status as a control, and age of death greater than 65. Autopsy diagnosis was performed by board certified neuropathologists and was based on the presence or absence of the characterization of probable or possible AD. Where it was possible, Braak and Braak staging and/or CERAD classification were employed. Samples derived from subjects with a clinical history of stroke, cerebrovascular disease, comorbidity with any other known neurological disease, or with the neuropathological finding of Lewy bodies were excluded.

**Mayo Clinic:** All 728 cases and 1,173 controls consisted of Caucasian subjects from the United States ascertained at the Mayo Clinic. All subjects were diagnosed by a neurologist at the Mayo Clinic in Jacksonville, Florida or Rochester, Minnesota. The neurologist confirmed a Clinical Dementia Rating score of 0 for all controls; cases had diagnoses of possible or probable AD made according to NINCDS-ADRDA criteria [9]. Autopsy-confirmed samples (221 cases, 216 CNEs) came from the brain bank at the Mayo Clinic in Jacksonville, FL and were evaluated by a single neuropathologist. In clinically-identified cases, the diagnosis of definite AD was made according to NINCDS-ADRDA criteria [9]. All AD brains analyzed in the study had a Braak score of 4.0 or greater. Brains employed as controls had a Braak score of 2.5 or lower but often had brain pathology unrelated to AD and pathological diagnoses that included vascular dementia, frontotemporal dementia, dementia with Lewy bodies, multi-system atrophy, amyotrophic lateral sclerosis, and progressive supranuclear palsy.

**The ROS/MAP Studies:** ROS/MAP are two community-based cohort studies. The ROS has been on-going since 1993, with a rolling admission. Through July of 2010, 1,139 older nuns, priests, and brothers from across the United States initially free of dementia who agreed to annual clinical evaluation and brain donation at the time of death completed their baseline evaluation. The MAP has been on-going since 1997, also with a rolling admission. Through July of 2010, 1,356 older persons from across northeastern Illinois initially free of dementia who agreed to annual clinical evaluation and organ donation at the time of death completed their baseline evaluation. Details of the clinical and neuropathologic evaluations have been previously reported [26, 27, 28, 29]. A total of 1,072 persons passed genotyping QC. Of these, 296 met clinical criteria for AD at the time of their last clinical evaluation or time of death and met neuropathologic criteria for AD for those on whom neuropathologic data were available, and 776 were without dementia or MCI at the time of their last clinical evaluation or time of death and did not meet neuropathologic criteria for AD for those on whom neuropathologic data were available.

**University of Pittsburgh (UP):** The University of Pittsburgh dataset contains 1,271 Caucasian AD cases (of which 277 were autopsy-confirmed) recruited by the University of Pittsburgh Alzheimer’s Disease Research Center, and 841 Caucasian, CNEs ages 60 and older (2 were autopsy-confirmed). All AD cases met NINCDS/ADRDA criteria for probable or definite AD. Additional details of the cohort used for GWAS have been previously published [30].

**Washington University (WU):** A European American LOAD case-control dataset consisting of 339 cases and 187 healthy elderly controls was used in analyses for this study. Participants were recruited as part of a longitudinal study of healthy aging and dementia. Diagnosis of dementia etiology was made in accordance with standard criteria and methods [3]. Severity of dementia was assessed using the Clinical Dementia Rating scale.

**The Cohort for Heart and Ageing Research in Genomic Epidemiology (CHARGE) consortium**

The CHARGE consortium currently includes six large, prospective, community-based cohort studies that have genome-wide variation data coupled with extensive data on multiple phenotypes [31]. A neurology working-group arrived at a consensus on phenotype harmonization, covariate selection and analytic plans for within-study analyses and meta-analysis of results [32]. Consent procedures, examination and surveillance components, data security, genotyping protocols and study design at each study were approved by a local Institutional Review Board, details are provided below. Of the six studies, two, the Atherosclerosis Risk in Communities (ARIC) study and the Austrian Study of Stroke Prevention (ASPS) study were excluded from these analyses as they had not systematically ascertained all their dementia cases at the time of this analysis. Three of the remaining 4 studies, the Cardiovascular Health Study (CHS), the Framingham Heart Study (FHS) and the Rotterdam Study had data on both the baseline prevalence, and on incident Alzheimer’s disease (AD), whereas the last, the Age, Gene/Environment Susceptibility– Reykjavik Study (AGES-RS) only had data on prevalent AD.

**AGES-RS:** The AGES–RS is a single center prospective study based on the Reykjavik Study, which was initiated in 1967 by the Icelandic Heart Association to study cardiovascular disease and risk factors. The cohort included men and women born 1907-35 and living in Reykjavik in 1967. In 2002, the National Institute of Aging in collaboration with the Icelandic Heart Association started the AGES study that focuses on 4 biological systems: vascular, neurocognitive, musculoskeletal and metabolism [33]. Between 2002 and 2006, it enrolled 5,764 participants (42% male) who were randomly selected among the survivors (n= 8,030) of the Reykjavik Study. All cohort members were European Caucasians. Participants were evaluated with a questionnaire and clinical exam, had a fasting sample of blood drawn, and underwent various bio-imaging measures. Of the 5,764 participants, 3,664 participants were randomly selected for the GWAS. Genotyping was undertaken using the HumanCNV370-Duo (Illumina) at the Laboratory of Neurogenetics, Intramural Research Program, at the National Institute of Aging, Bethesda, Maryland. A total of 2,807 persons passed genotyping QC criteria, and, based on the methods outlined in Section 3, were categorized as either non-demented or having Alzheimer’s disease (AD). The AGES-RS was approved by the Icelandic National Bioethics Committee (VSN 00-063), the Icelandic Data Protection Authority, and by the Institutional Review Board of the US National Institute on Aging, National Institutes of Health. This study included all persons with prevalent AD detected between 2002 and 2006. The Folstein Mini Mental State Examination (MMSE) and the Digit Symbol Substitution Test (DSST) were administered to all participants and persons who scored below a pre-determined threshold on these tests (≤23 on the MMSE or ≤17 on the DSST) were administered a second, diagnostic test battery. Based on performance on the Trails B and the Rey Auditory Verbal Learning test (RAVLT), a subset of these individuals with a RAVLT score ≤18 or Trails B score ≥8 (ratio of time taken for Trails B/Trails A corrected for the number correct) went on to a third step, which included a neurological examination and a structured informant interview about medical history and social, cognitive, and daily functioning. MRI was acquired as a part of the core study protocol. A panel that included a geriatrician, neurologist, neuropsychologist, and neuroradiologist reached a consensus diagnosis of dementia based on the Diagnostic and Statistical Manual of Mental Disorders, Fourth Edition (DSM-IV) guidelines. There were 319 cases of dementia diagnosed in the first 5,764 AGES participants and of these 123 also had genotyping and brain MRI. International diagnostic guidelines, including the National Institute of Neurological and Communicative Disorders and Stroke–Alzheimer Disease and Related Disorders Association (NINCDS-ADRDA) criteria for probable and possible Alzheimer Disease and the Alzheimer’s Disease Diagnosis and Treatment Center’s (ADDTC) State of California criteria for probable and possible vascular dementia (VaD) with or without AD, were followed. The AGES study identified 3 subtypes: possible/probable AD without VaD (n=55, included in analysis), mixed AD (n=23, cases that met criteria for both AD and VaD, also included in analysis), and, possible/probable VaD or other dementia without AD (n=45, excluded from this study).

**Cardiovascular Health Study (CHS):** The CHS is a prospective population-based cohort study of risk factors for vascular and metabolic disease that in 1989-90, enrolled adults aged ≥65 years, at four field centers located in North Carolina, California, Maryland and Pennsylvania [34]. The original predominantly Caucasian cohort of 5,201 persons was recruited from a random sample of people on Medicare eligibility lists and an additional 687 African-Americans were enrolled subsequently for a total sample of 5,888 [34]. Deoxyribonucleic acid (DNA) was extracted from blood samples drawn on all persons who consented to genetic testing at their baseline examination in 1989-90. In 2007-2008, genotyping was performed at the General Clinical Research Center’s Phenotyping/Genotyping Laboratory at Cedars-Sinai using the Illumina 370CNV Duo ® BeadChip system on 3,980 CHS participants who were free of cardiovascular disease (CVD) at baseline. The 1,908 persons excluded for prevalent CVD had prevalent coronary heart disease (n=1,195), congestive heart failure (n=86), peripheral vascular disease (n=93), valvular heart disease (n=20), stroke (n=166) or transient ischemic attack (n=56). Some persons had more than one reason to be excluded and for these individuals only the initial exclusionary event is listed. Because the other cohorts were predominantly white, the African American CHS participants were excluded from this analysis to limit errors secondary to population stratification. Among white participants genotyping was attempted in 3,397 participants and was successful in 3295 persons. A total of 742 persons were excluded as they either died prior to the start of the CHS cognition study in 1992 (see section 3 for details), or could not be evaluated completely for baseline cognitive status, leaving a baseline sample of 2,553 persons; an additional 31 persons were excluded for having dementia other than AD leaving a study sample of 2,522 persons. The CHS study protocols were approved by the Institutional review boards at the individual participating centers.

The AD sample for this study included all prevalent cases identified in 1992 and incident events identified between 1992 and December 2006 [35]. Briefly, persons were examined annually from enrollment to 1999, and the examination included a 30 minute screening cognitive battery. In 1992-94 and again, in 1997-99, participants were invited to undergo brain MRI and detailed cognitive and neurological assessment as part of the CHS Cognition Study. Persons with prevalent dementia were identified, and all others were followed until 1999 for the development of incident dementia and AD. Since then, CHS participants at the Maryland and Pennsylvania centers have remained under ongoing dementia surveillance [36].

Beginning in 1988/89, all participants completed the Modified Mini-Mental State Examination (3MSE) and the DSST at their annual visits, and the Benton Visual Retention Test (BVRT) from 1994 to 1998. The Telephone Interview for Cognitive Status (TICS) was used when participants did not come to the clinic. Further information on cognition was obtained from proxies using the Informant Questionnaire for Cognitive Decline in the Elderly (IQCODE), and the dementia questionnaire (DQ). Symptoms of depression were measured with the modified version of the Center for Epidemiology Studies Depression Scale (CES-D). In 1991-94, 3,608 participants had an MRI of the brain and this was repeated in 1997-98. The CHS staff also obtained information from participants and next-of-kin regarding vision and hearing, the circumstances of the illness, history of dementia, functional status, pharmaceutical drug use, and alcohol consumption. Data on instrumental activities of daily living (IADL), and activities of daily living (ADL) were also collected.

Persons suspected to have cognitive impairment based on the screening tests listed above underwent a neuropsychological and a neurological evaluation. The neuropsychological battery included the following tests: the American version of the National Reading test (AMNART), Raven’s Coloured Progressive Matrices, California Verbal Learning Test (CVLT), a modified Rey-Osterreith figure, the Boston Naming test, the Verbal fluency test, the Block design test, the Trails A and B tests, the Baddeley & Papagno Divided Attention Task, the Stroop, Digit Span and Grooved Pegboard Tests. The results of the neuropsychological battery were classified as normal or abnormal (>1.5 standard deviations below individuals of comparable age and education) based on normative data collected from a sample of 250 unimpaired subjects. The neurological exam included a brief mental status examination, as well as a complete examination of other systems. The examiner also completed the Unified Parkinson’s Disease Rating Scale (UPDRS) and the Hachinski Ischemic Scale. After completing the neurological exam, the neurologist classified the participant as normal, having mild cognitive impairment (MCI), or dementia. International diagnostic guidelines, including the NINCDS-ADRDA criteria for probable and possible AD and the ADDTC’s State of California criteria for probable and possible vascular dementia (VaD) with or without AD, were followed. CHS identified 3 subtypes: possible/probable AD without VaD (categorized as pure AD, included in all AD) and mixed AD (for cases that met criteria for both AD and VaD, included in all-AD), and, possible/probable VaD without AD (excluded from current study).

**Framingham Heart Study (FHS):** The FHS is a three-generation, single-site, community-based, ongoing cohort study that was initiated in 1948. It now comprises three generations of participants including the Original cohort followed since 1948 (n=5,209) [37], their Offspring and spouses of the offspring (n=5,216) followed since 1971 [38]; and children from the largest Offspring families enrolled in 2000 (Gen 3) [39]. Participants in the Original and Offspring cohorts are used in these analyses, but Gen 3 participants were not included since they are young (mean age 40±9 years in 2000) and none had developed Alzheimer’s Disease (AD). The Original cohort enrolled 5,209 men and women who comprised two-thirds of the adult population then residing in Framingham, Massachusetts. Survivors continue to receive biennial examinations. The Offspring cohort comprises 5,124 persons (including 3,514 biological offspring) who have been examined approximately once every 4 years. Almost all the FHS Original and Offspring participants are white/Caucasian. FHS participants had DNA extracted and provided consent for genotyping in the 1990s. All available eligible participants were genotyped at Affymetrix (Santa Clara, CA) through an NHLBI funded SNP-Health Association Resource (SHARe) project using the Affymetrix GeneChip® Human Mapping 500K Array Set and 50K Human Gene Focused Panel®. In 272 persons, small amounts of DNA were extracted from stored whole blood and required whole genome amplification prior to genotyping. Cell lines were available for most of the remaining participants. Genotyping was attempted in 5,293 Original and Offspring cohort participants, and 4,425 persons met QC criteria. Failures (call rate <97%, extreme heterozygosity or high Mendelian error rate) were largely restricted to persons with whole-genome amplified DNA and DNA extracted from stored serum samples. In addition, since the persons with whole genome amplified DNA represent a group of survivors who may di_er from the others we included whole genome amplified status as a covariate in FHS analyses. For the prevalent analyses, we also excluded 2,268 participants who were less than 65 years old at the time of the DNA draw and 14 persons with dementia other than AD; the remaining 2,143 subjects constitute the FHS sample for the prevalent study. A total of 806 well-genotyped persons from the Original cohort (which has been under ongoing surveillance for incident dementia since 1975) were included in the incident AD analyses. The FHS component of this study was approved by the Institutional Review Board of the Boston Medical Center.

The Original cohort of the FHS has been evaluated biennially since 1948, was screened for prevalent dementia and AD in 1974-76 and has been under surveillance for incident dementia and AD since then [40, 41, 42]. The Offspring have been examined once every 4 years and have been screened for prevalent dementia with a neuropsychological battery and brain MRI [43, 44]. In order to be consistent with the sampling frame for the AGES and CHS samples, we excluded FHS subjects with a baseline age <65 yrs at the time of DNA draw which was in the 1990s. To minimize survival biases, Original cohort and O_spring participants who developed dementia prior to the date of DNA draw were treated as prevalent cases, and subsequent events in the Original cohort occurring prior to December 2006 were included in the incident analyses.

At each clinic exam, participants receive questionnaires, physical examinations and laboratory testing; between examinations they remain under surveillance (regardless of whether or not they live in the vicinity) via physician referrals, record linkage and annual telephone health history updates. Methods used for dementia screening and follow-up have been previously described [40, 45]. Briefly, surviving cohort members who attended biennial examination cycles 14 and 15 (May 1975-November 1979) were administered a standardized neuropsychological test battery to establish a dementia-free cohort. Beginning at examination cycle 17 (1982), the MMSE was administered biennially to the cohort. A MMSE score below the education-specific cutoff score, a decline of 3 or more points on subsequent administrations, a decline of more than 5 points compared with any previous examination, or a physician or family referral prompted further in-depth testing. The Offspring cohort that was enrolled in 1971 has undergone 8 re-examinations, one approximately every 4 years. Starting at the 2nd Offspring examination, participants were questioned regarding any subjective memory complaints and since the 5th Offspring examination participants have been administered the MMSE at each visit. In addition concurrent with the 7th and 8th Offspring examinations (between 1999 and 2004 and then again between 2005 and 2009) surviving Original cohort and all eligible and consenting Offspring participants have undergone volumetric brain MRI and neuropsychological testing [43, 44]. The neuropsychological test battery included the Reading subtest of the Wide Range Achievement Test (WRAT-3), the Logical Memory and the Paired Associates Learning tests from the Wechsler Memory Scale, the Visual Reproduction and Hooper Visual Organization Tests, Trails A and B, the Similarities subtest from the Wechsler Adult Intelligence test, the 30-iterm version of the Boston Naming Test and at the second assessment only, the Digit Span, Controlled Word Association and Clock Drawing Tests. Offspring participants suspected to have cognitive impairment based on their MMSE scores, participant, family or physician referral, hospital records

or performance in the neuropsychological test battery described above were referred for more detailed neuropsy-chological and neurological evaluation.

Each participant thus identified underwent baseline neurologic and neuropsychological examinations. Neurologists (trained in geriatric behavioral assessment) supplemented their clinical assessment with a few structured cognitive tests and administered the Clinical Dementia Rating (CDR). Persons were reassessed systematically for the onset of at least mild dementia. A panel consisting of at least 1 neurologist (S.A., P.A.W., or S.S.) and 1 neuropsychologist (R.A.) reviewed all available medical records to arrive at a final determination regarding the presence or absence of dementia, the date of onset of dementia, and the type of dementia. For this determination, we used data from the neurologist’s examination, neuropsychological test performance, Framingham Study records, hospital records, information from primary care physicians, structured family interviews, computed tomography and magnetic resonance imaging records, and autopsy confirmation when available. All individuals identified as having dementia satisfied the DSM-IV criteria, had dementia severity equivalent to a CDR of 1 or greater, and had symptoms of dementia for at least 6 months. All individuals identified as having Alzheimer-related dementia met the NINCDS-ADRDA criteria for definite, probable, or possible AD. Vascular Dementia was diagnosed using the ADDTC criteria but the presence of vascular dementia did not disqualify a participant from obtaining a concomitant diagnosis of AD if indicated. The recruitment of Original cohort participants at FHS had occurred long before the DNA collection with the result that the majority of dementia events in the FHS (although ascertained prospectively) were prevalent at the time of DNA collection or these persons had died prior to DNA draw and were thus excluded from analyses of incident disease. Due to the limited number of incident dementia and AD events in the Framingham Offspring only the Original cohort were included in our analyses of incident events.

**Rotterdam Study:** The Rotterdam Study enrolled inhabitants from a district of Rotterdam (Ommoord) aged ≥55 years (N=7,983, virtually all white) at the baseline examination in 1990-93 when blood was drawn for genotyping [46, 47]. It aims to examine the determinants of disease and health in the elderly with a focus on neurogeriatric, cardiovascular, bone, and eye disease [8]. All inhabitants of Ommoord aged ≥55 years (n = 10,275) were invited and the participation rate was 78%. All participants gave written informed consent to retrieve information from treating physicians. Baseline measurements were obtained from 1990 to 1993 and consisted of an interview at home and two visits to the research center for physical examination. Survivors have been re-examined three times: in 1993-1995, 1997-1999, and 2002-2004. All persons attending the baseline examination in 1990-93 consented to

genotyping and had DNA extracted. This DNA was genotyped using the Illumina Infinium II HumanHap550chip v3·0® array in 2007-2008 according to the manufacturer’s protocols. Genotyping was attempted in persons with high-quality extracted DNA (n=6,449). From these 6,449, samples with low call rate (<97.5%, n=209), with excess autosomal heterozygosity (>0.336, n=21), with sex-mismatch (n=36), or if there were outliers identified by the IBS clustering analysis (>3 standard deviations from population mean, n=102 or IBS probabilities >97%, n=129) were excluded from the study population with some persons meeting more than one exclusion criterion; in total, 5,974 samples were available with good quality genotyping data, 42 persons were excluded since they did not undergo cognitive screening at baseline, hence their cognitive status was uncertain. An additional 61 persons were excluded because they suffered from dementia other than AD at baseline. Thus there were 5,871 persons included in the prevalent analysis and after exclusion of 171 persons with prevalent AD, 5,700 persons were followed for incident AD and other dementia. The Rotterdam Study (including its brain magnetic resonance imaging (MRI) and neurological components) has been approved by the institutional review board (Medical Ethics Committee) of the Erasmus Medical Center and the Netherlands Ministry of Health, Welfare and Sports Participants were screened for prevalent dementia in 1990-93 using a three-stage process; those free of dementia remained under surveillance for incident dementia, a determination made using records linkage and assessment at three subsequent re-examinations [48]. We included all prevalent cases and all incident events up to 31st December 2007.

Screening was done with the MMSE and GMS organic level for all persons. Screen-positives (MMSE <26 or Geriatric Mental Schedule (GMS) organic level >0) underwent the CAMDEX. Persons who were suspected of having dementia underwent more extensive neuropsychological testing. When available, imaging data were used. In addition, all participants have been continuously monitored for major events (including dementia) through automated linkage of the study database with digitized medical records from general practitioners, the Regional Institute for Outpatient Mental Health Care and the municipality. In addition physician files from nursing homes and general practitioner records of participants who moved out of the Ommoord district were reviewed twice a year. For suspected dementia events, additional information (including neuroimaging) was obtained from hospital records and research physicians discussed available information with a neurologist experienced in dementia diagnosis and research to verify all diagnoses. Dementia was diagnosed in accordance with internationally accepted criteria for dementia (Diagnostic and Statistical Manual of Mental Disorders, Revised Third Edition, DSM-III-R), and AD using the NINCDS-ADRDA criteria for possible, probable and definite AD. The National Institute of Neurological Disorders and Stroke–Association Internationale pour la Recherche et l’Enseignement en Neurosciences (NINDSAIREN) criteria were used to diagnose vascular dementia. The final diagnosis was determined by a panel of a neurologist, neurophysiologist, and research physician and the diagnoses of AD and VaD were not mutually exclusive.

**European Alzheimer’s Disease Initiative (EADI) consortium**

All the 2,243 AD cases were ascertained by neurologists from Bordeaux, Dijon, Lille, Montpellier, Paris, Rouen, and were identified as French Caucasian [49, 50]. Clinical diagnosis of probable AD was established according to the DSM-III-R and NINCDS-ADRDA criteria. Controls were selected from the 3C Study [50]. This cohort is a population-based, prospective (7-years follow-up) study of the relationship between vascular factors and dementia. It has been carried out in three French cities: Bordeaux (southwest France), Montpellier (southeast France) and Dijon (central eastern France). A sample of non-institutionalised, over-65 subjects was randomly selected from the electoral rolls of each city. Between January 1999 and March 2001, 9,686 subjects meeting the inclusion criteria agreed to participate. Following recruitment, 392 subjects withdrew from the study. Thus, 9,294 subjects were finally included in the study (2,104 in Bordeaux, 4,931 in Dijon and 2,259 in Montpellier). Genomic DNA samples of 7,200 individuals were transferred to the French Centre National de Génotypage (CNG). First stage samples that passed DNA quality control were genotyped with Illumina Human 610-Quad BeadChips. At the end we removed 308 samples because they were found to be first- or second-degree relatives of other study participants, or were assessed non-European descent based on genetic analysis using methods described in [51]. In this final sample, at 7 years of follow-up, 459 individuals su_ered from AD with 97 prevalent and 362 incident cases. These AD cases were included as cases in the EADI stage 1 dataset. We retained the other individuals as controls (n=6,017).

**Genetic and Environmental Risk in Alzheimer’s Disease (GERAD) consortium**

The GERAD sample comprises 3,177 AD cases and 7,277 controls with available age and gender data [52]. Cases and elderly screened controls were recruited by the Medical Research Council (MRC) Genetic Resource for AD (Cardiff University; Institute of Psychiatry, London; Cambridge University; Trinity College Dublin), the Alzheimer’s Research Trust (ART) Collaboration (University of Nottingham; University of Manchester; University of Southampton; University of Bristol; Queen’s University Belfast; the Oxford Project to Investigate Memory and Ageing (OPTIMA), Oxford University); Washington University, St Louis, United States; MRC PRION Unit, University College London; London and the South East Region AD project (LASER-AD), University College London; Competence Network of Dementia (CND) and Department of Psychiatry, University of Bonn, Germany; the National Institute of Mental Health (NIMH) AD Genetics Initiative. 6,129 population controls were drawn from large existing cohorts with available GWAS data, including the 1958 British Birth Cohort (1958BC) (http://www.b58cgene.sgul.ac.uk), the KORA F4 Study and the Heinz Nixdorf Recall Study. All AD cases met criteria for either probable (NINCDS-ADRDA, DSM-IV) or definite (CERAD) AD All elderly controls were screened for dementia using the MMSE or ADAS-cog, were determined to be free from dementia at neuropathological examination or had a Braak score of 2.5 or lower. Genotypes from all cases and 4,617 controls were previously included in the AD GWAS by Harold and colleagues [52]. Genotypes for the remaining 2,660 population controls were obtained from WTCCC2.

**Stage 2 case-control studies**

Additional 22,618 case-control samples were obtained for replication from centres in Austria (1 centre), Belgium (1 centre), Finland (1 centre), Germany (4 centres), Greece (1 centre), Hungary (1 centre), Italy (8 centres), Spain (7 centres), Sweden (2 centres), the UK (5 centres) and the USA (1 centre). Clinical diagnoses of probable AD were all established according to the DSM-III-R and NINCDS-ADRDA criteria. Controls were defined as subjects without DMS-III-R dementia criteria and with integrity of their cognitive functions (MMS >25). Each laboratory was asked to send 2.5 µg of genomic DNA at 50 ng/µl.

Genotyping of ASPS Austrian control samples was performed using the Illumina Human610-Quad BeadChip. We excluded samples with low call rate (<98%), with excess autosomal heterozygosity (>0.350), with sex-mismatch, or as outliers identified by the IBS clustering analysis (>3 standard deviations from population mean or IBS probabilities >97%). Imputation was done using 1000G phase 1 interim (June 2011) data with the IMPUTE2 software. German individuals from Bonn centre were genotyped using the Illumina Omni1-Quad chip. Individuals with a genotype call rate worse than 99% or identified as outliers by a PCA were excluded. Identical DNAs and relationships between probands were checked by computation of the IBS-matrix and we removed one individual from each pair with an IBS that was 4 standard deviations higher than the average IBS of all pairs of individuals. Some of the AD patients were part of the original GERAD study. All doubled individuals were identified via IBS-analysis and were excluded. Imputation was performed with IMPUTE2 using 1,000 Genomes reference data (Feb 2012 release).

**References**

[1] Jun G, Naj AC, Beecham GW, Wang LS, Buros J, Gallins PJ, et al. Meta-analysis confirms CR1, CLU, and PICALM as alzheimer disease risk loci and reveals interactions with APOE genotypes. Arch Neurol. 2010 Dec;67(12):1473–1484.

[2] Beekly DL, Ramos EM, Lee WW, Deitrich WD, Jacka ME, Wu J, et al. The National Alzheimer’s Coordinating Center (NACC) database: the Uniform Data Set. Alzheimer Dis Assoc Disord. 2007;21(3):249–258.

[3] Morris JC, Weintraub S, Chui HC, Cummings J, Decarli C, Ferris S, et al. The Uniform Data Set (UDS): clinical and cognitive variables and descriptive data from Alzheimer Disease Centers. Alzheimer Dis Assoc Disord. 2006;20(4):210–216.

[4] Hyman BT, Trojanowski JQ. Consensus recommendations for the postmortem diagnosis of Alzheimer disease from the National Institute on Aging and the Reagan Institute Working Group on diagnostic criteria for the neuropathological assessment of Alzheimer disease. J Neuropathol Exp Neurol. 1997 Oct;56(10):1095–1097.

[5] Mirra SS, Hart MN, Terry RD. Making the diagnosis of Alzheimer’s disease. A primer for practicing pathologists. Arch Pathol Lab Med. 1993 Feb;117(2):132–144.

[6] Nagy Z, Yilmazer-Hanke DM, Braak H, Braak E, Schultz C, Hanke J. Assessment of the pathological stages of Alzheimer’s disease in thin para_n sections: a comparative study. Dement Geriatr Cogn Disord. 1998;9(3):140–144.

[7] Braak H, Braak E. Neuropathological stageing of Alzheimer-related changes. Acta Neuropathol. 1991;82(4):239–259.

[8] Kramer PL, Xu H,Woltjer RL,Westaway SK, Clark D, Erten-Lyons D, et al. Alzheimer disease pathology in cognitively healthy elderly: a genome-wide study. Neurobiol Aging. 2011 Dec;32(12):2113–2122.

[9] McKhann G, Drachman D, Folstein M, Katzman R, Price D, Stadlan EM. Clinical diagnosis of Alzheimer’s disease: report of the NINCDS-ADRDAWork Group under the auspices of Department of Health and Human Services Task Force on Alzheimer’s Disease. Neurology. 1984 Jul;34(7):939–944.

[10] Petersen RC, Aisen PS, Beckett LA, Donohue MC, Gamst AC, Harvey DJ, et al. Alzheimer’s Disease Neuroimaging Initiative (ADNI): clinical characterization. Neurology. 2010 Jan;74(3):201–209.

[11] Saykin AJ, Shen L, Foroud TM, Potkin SG, Swaminathan S, Kim S, et al. Alzheimer’s Disease Neuroimaging Initiative biomarkers as quantitative phenotypes: Genetics core aims, progress, and plans. Alzheimers Dement. 2010 May;6(3):265–273.

[12] Green RC, Cupples LA, Go R, Benke KS, Edeki T, Griffth PA, et al. Risk of dementia among white and African American relatives of patients with Alzheimer disease. JAMA. 2002 Jan;287(3):329–336.

[13] Roccaforte WH, Burke WJ, Bayer BL, Wengel SP. Validation of a telephone version of the mini-mental state examination. J Am Geriatr Soc. 1992 Jul;40(7):697–702.

[14] Lee JH, Cheng R, Gra_-Radford N, Foroud T, Mayeux R, on Aging Late-Onset Alzheimer’s Disease Family Study Group NI. Analyses of the National Institute on Aging Late-Onset Alzheimer’s Disease Family Study: implication of additional loci. Arch Neurol. 2008 Nov;65(11):1518–1526.

[15] Scott WK, Nance MA, Watts RL, Hubble JP, Koller WC, Lyons K, et al. Complete genomic screen in Parkinson disease: evidence for multiple genes. JAMA. 2001 Nov;286(18):2239–2244.

[16] Beecham GW, Martin ER, Li YJ, Slifer MA, Gilbert JR, Haines JL, et al. Genome-wide association study implicates a chromosome 12 risk locus for late-onset Alzheimer disease. Am J Hum Genet. 2009 Jan;84(1):35–43.

[17] Edwards TL, Scott WK, Almonte C, Burt A, Powell EH, Beecham GW, et al. Genome-wide association study confirms SNPs in SNCA and the MAPT region as common risk factors for Parkinson disease. Ann Hum Genet. 2010 Mar;74(2):97–109.

[18] Naj AC, Beecham GW, Martin ER, Gallins PJ, Powell EH, Konidari I, et al. Dementia revealed: novel chromosome 6 locus for late-onset Alzheimer disease provides genetic evidence for folate-pathway abnormalities. PLoS Genet. 2010 Sep;6(9).

[19] Haroutunian V, Perl DP, Purohit DP, Marin D, Khan K, Lantz M, et al. Regional distribution of neuritic plaques in the nondemented elderly and subjects with very mild Alzheimer disease. Arch Neurol. 1998 Sep;55(9):1185–1191.

[20] KukullWA, Higdon R, Bowen JD, McCormick WC, Teri L, Schellenberg GD, et al. Dementia and Alzheimer disease incidence: a prospective cohort study. Arch Neurol. 2002 Nov;59(11):1737–1746.

[21] Larson EB, Wang L, Bowen JD, McCormick WC, Teri L, Crane P, et al. Exercise is associated with reduced risk for incident dementia among persons 65 years of age and older. Ann Intern Med. 2006 Jan;144(2):73–81.

[22] Li H, Wetten S, Li L, St Jean PL, Upmanyu R, Surh L, et al. Candidate single-nucleotide polymorphisms from a genomewide association study of Alzheimer disease. Arch Neurol. 2008 Jan;65(1):45–53.

[23] Reiman EM, Webster JA, Myers AJ, Hardy J, Dunckley T, Zismann VL, et al. GAB2 alleles modify Alzheimer’s risk in APOE epsilon4 carriers. Neuron. 2007 Jun;54(5):713–720.

[24] Caselli RJ, Reiman EM, Locke DEC, Hutton ML, Hentz JG, Hoffman-Snyder C, et al. Cognitive domain decline in healthy apolipoprotein E epsilon4 homozygotes before the diagnosis of mild cognitive impairment. Arch Neurol. 2007 Sep;64(9):1306–1311.

[25] Webster JA, Gibbs JR, Clarke J, Ray M, ZhangW, Holmans P, et al. Genetic control of human brain transcript expression in Alzheimer disease. Am J Hum Genet. 2009 Apr;84(4):445–458.

[26] Bennett DA, Wilson RS, Schneider JA, Evans DA, Beckett LA, Aggarwal NT, et al. Natural history of mild cognitive impairment in older persons. Neurology. 2002 Jul;59(2):198–205.

[27] Bennett DA, Schneider JA, Bienias JL, Evans DA, Wilson RS. Mild cognitive impairment is related to Alzheimer disease pathology and cerebral infarctions. Neurology. 2005 Mar;64(5):834–841.

[28] Bennett DA, Schneider JA, Buchman AS, de Leon CM, Bienias JL,Wilson RS. The Rush Memory and Aging Project: study design and baseline characteristics of the study cohort. Neuroepidemiology. 2005;25(4):163–175.

[29] Schneider JA, Arvanitakis Z, BangW, Bennett DA. Mixed brain pathologies account for most dementia cases in community-dwelling older persons. Neurology. 2007 Dec;69(24):2197–2204.

[30] Kamboh MI, Minster RL, Demirci FY, Ganguli M, Dekosky ST, Lopez OL, et al. Association of CLU and PICALM variants with Alzheimer’s disease. Neurobiol Aging. 2012 Mar;33(3):518–521.

[31] Psaty BM, O’Donnell CJ, Gudnason V, Lunetta KL, Folsom AR, Rotter JI, et al. Cohorts for Heart and Aging Research in Genomic Epidemiology (CHARGE) Consortium: Design of prospective meta-analyses of genome-wide association studies from 5 cohorts. Circ Cardiovasc Genet. 2009 Feb;2(1):73–80.

[32] Ikram MA, Seshadri S, Bis JC, Fornage M, DeStefano AL, Aulchenko YS, et al. Genomewide association studies of stroke. N Engl J Med. 2009 Apr;360(17):1718–1728.

[33] Harris TB, Launer LJ, Eiriksdottir G, Kjartansson O, Jonsson PV, Sigurdsson G, et al. Age, Gene/Environment Susceptibility-Reykjavik Study: multidisciplinary applied phenomics. Am J Epidemiol. 2007 May;165(9):1076–1087.

[34] Fried LP, Borhani NO, Enright P, Furberg CD, Gardin JM, Kronmal RA, et al. The Cardiovascular Health Study: design and rationale. Ann Epidemiol. 1991 Feb;1(3):263–276.

[35] Fitzpatrick AL, Kuller LH, Ives DG, Lopez OL, Jagust W, Breitner JCS, et al. Incidence and prevalence of dementia in the Cardiovascular Health Study. J Am Geriatr Soc. 2004 Feb;52(2):195–204.

[36] Lopez OL, Kuller LH, Fitzpatrick A, Ives D, Becker JT, Beauchamp N. Evaluation of dementia in the cardiovascular health cognition study. Neuroepidemiology. 2003;22(1):1–12.

[37] Dawber TR, Kannel WB. The Framingham study. An epidemiological approach to coronary heart disease. Circulation. 1966 Oct;34(4):553–555.

[38] Feinleib M, Kannel WB, Garrison RJ, McNamara PM, Castelli WP. The Framingham O_spring Study. Design and preliminary data. Prev Med. 1975 Dec;4(4):518–525.

[39] Splansky GL, Corey D, Yang Q, Atwood LD, Cupples LA, Benjamin EJ, et al. The Third Generation Cohort of the National Heart, Lung, and Blood Institute’s Framingham Heart Study: design, recruitment, and initial examination. Am J Epidemiol. 2007 Jun;165(11):1328–1335.

[40] Beiser A, D’Agostino RB, Seshadri S, Sullivan LM, Wolf PA. Computing estimates of incidence, including lifetime risk: Alzheimer’s disease in the Framingham Study. The Practical Incidence Estimators (PIE) macro. Stat Med. 2000;19(11-12):1495–1522.

[41] Bachman DL, Wolf PA, Linn RT, Knoefel JE, Cobb JL, Belanger AJ, et al. Incidence of dementia and probable Alzheimer’s disease in a general population: the Framingham Study. Neurology. 1993 Mar;43(3 Pt 1):515–519.

[42] Farmer ME, White LR, Kittner SJ, Kaplan E, Moes E, McNamara P, et al. Neuropsychological test performance in Framingham: a descriptive study. Psychol Rep. 1987 Jun;60(3 Pt 2):1023–1040.

[43] DeCarli C, Massaro J, Harvey D, Hald J, Tullberg M, Au R, et al. Measures of brain morphology and infarction in the framingham heart study: establishing what is normal. Neurobiol Aging. 2005 Apr;26(4):491–510.

[44] Au R, Seshadri S, Wolf PA, Elias M, Elias P, Sullivan L, et al. New norms for a new generation: cognitive performance in the framingham o_spring cohort. Exp Aging Res. 2004;30(4):333–358.

[45] Seshadri S, Wolf PA, Beiser A, Au R, McNulty K, White R, et al. Lifetime risk of dementia and Alzheimer’s disease. The impact of mortality on risk estimates in the Framingham Study. Neurology. 1997 Dec;49(6):1498–1504.

[46] Hofman A, Breteler MMB, van Duijn CM, Janssen HLA, Krestin GP, Kuipers EJ, et al. The Rotterdam Study: 2010 objectives and design update. Eur J Epidemiol. 2009;24(9):553–572.

[47] Hofman A, Breteler MMB, van Duijn CM, Krestin GP, Pols HA, Stricker BHC, et al. The Rotterdam Study: objectives and design update. Eur J Epidemiol. 2007;22(11):819–829.

[48] Ott A, Breteler MM, van Harskamp F, Stijnen T, Hofman A. Incidence and risk of dementia. The Rotterdam Study. Am J Epidemiol. 1998 Mar;147(6):574–580.

[49] Dreses-Werringloer U, Lambert JC, Vingtdeux V, Zhao H, Vais H, Siebert A, et al. A polymorphism in CALHM1 influences Ca2+ homeostasis, Abeta levels, and Alzheimer’s disease risk. Cell. 2008 Jun;133(7):1149–1161.

[50] 3C Study Group. Vascular factors and risk of dementia: design of the Three-City Study and baseline characteristics of the study population. Neuroepidemiology. 2003;22(6):316–325.

[51] Heath SC, Gut IG, Brennan P, McKay JD, Bencko V, Fabianova E, et al. Investigation of the fine structure

of European populations with applications to disease association studies. Eur J Hum Genet. 2008 Dec;16(12):1413–1429.

[52] Harold D, Abraham R, Hollingworth P, Sims R, Gerrish A, Hamshere ML, et al. Genome-wide association

study identifies variants at CLU and PICALM associated with Alzheimer’s disease. Nat Genet. 2009 Oct;41(10):1088–1093.

[53] Mangravite LM, Engelhardt BE, et al. Bayes Factor, LCLs, 480 ids from Cholesterol And Pharmacogenetics (CAP) study. (in review);.

[54] Zeller T, Wild P, Szymczak S, Rotival M, Schillert A, Castagne R, et al. Genetics and beyond–the transcriptome of human monocytes and disease susceptibility. PLoS One. 2010;5(5):e10693.

[55] Montgomery SB, Sammeth M, Gutierrez-Arcelus M, Lach RP, Ingle C, Nisbett J, et al. Transcriptome genetics using second generation sequencing in a Caucasian population. Nature. 2010 Apr;464(7289):773–777.

[56] Stranger BE, Nica AC, Forrest MS, Dimas A, Bird CP, Beazley C, et al. Population genomics of human gene expression. Nat Genet. 2007 Oct;39(10):1217–1224.

[57] Veyrieras JB, Kudaravalli S, Kim SY, Dermitzakis ET, Gilad Y, Stephens M, et al. High-resolution mapping of expression-QTLs yields insight into human gene regulation. PLoS Genet. 2008 Oct;4(10):e1000214.
