# Supplementary material for: Gene-Wide Analysis Detects Two New Susceptibility Genes for Alzheimer's Disease
Source: PLoS One. 2014 Jun 12;9(6):e94661. doi: 10.1371/journal.pone.0094661 (PMC4055488; doi:10.1371/journal.pone.0094661)
Supplement: Materials S3 — Acknowledgements. (DOCX) [file pone.0094661.s019.docx]

**Acknowledgements**

This work was made possible by the generous participation of the control subjects, the patients, and their families. The i-Select chips was funded by the French National Foundation on Alzheimer’s disease and related disorders. The French National Fondation on Alzheimer’s disease and related disorders supported several I-GAP meetings and communications. Data management involved the Centre National de Génotypage,and was supported by the Institut Pasteur de Lille, Inserm, FRC (fondation pour la recherche sur le cerveau) and Rotary. This work has been developed and supported by the LABEX (laboratory of excellence program investment for the future) DISTALZ grant (Development of Innovative Strategies for a Transdisciplinary approach to ALZheimer’s disease) and by the LABEX GENMED grant (Medical Genomics). The French National Foundation on Alzheimer’s disease and related disorders and the Alzheimer’s Association (Chicago, IL) grant supported IGAP in-person meetings, communication and the Alzheimer’s Association (Chicago, IL) grant provided some funds to each consortium for analyses.

**EADI**

We thank Dr. Anne Boland (CNG) for her technical help in preparing the DNA samples for analyses. This work was supported by the National Foundation for Alzheimer’s disease and related disorders, the Institut Pasteur de Lille and the Centre National de Génotypage.

The Three-City Study was performed as part of a collaboration between the Institut National de la Santé et de la Recherche Médicale (Inserm), the Victor Segalen Bordeaux II University and Sanofi-Synthélabo. The Fondation pour la Recherche Médicale funded the preparation and initiation of the study. The 3C Study was also funded by the Caisse Nationale Maladie des Travailleurs Salariés, Direction Générale de la Santé, MGEN, Institut de la Longévité, Agence Française de Sécurité Sanitaire des Produits de Santé, the Aquitaine and Bourgogne Regional Councils, Agence Nationale de la Recherche, ANR supported the COGINUT and COVADIS projects. Fondation de France and the joint French Ministry of Research/INSERM «Cohortes et collections de données biologiques» programme. Lille Génopôle received an unconditional grant from Eisai. The Three-city biological bank was developed and maintained by the laboratory for genomic analysis LAG-BRC - Institut Pasteur de Lille.

Belgium sample collection: The patients were clinically and pathological characterized by the neurologists Sebastiaan Engelborghs, Rik Vandenberghe and Peter P De Deyn, and in part genetically by Caroline Van Cauwenberghe, Karolien Bettens and Kristel Sleegers. Research at the Antwerp site is funded in part by the Belgian Science Policy Offce Interuniversity Attraction Poles program, the Foundation Alzheimer Research (SAO-FRA), the Flemish Government initiated Methusalem Excellence Program, the Research Foundation Flanders (FWO) and the University of Antwerp Research Fund, Belgium. KB is a postdoctoral fellow of the FWO. The Antwerp site authors thank the personnel of the VIB Genetic Service Facility, the Biobank of the Institute Born-Bunge and the Departments of Neurology and Memory Clinics at the Hospital Network Antwerp and the University Hospitals Leuven.

Finish sample collection : Financial support for this project was provided by the Health Research Council of the Academy of Finland, EVO grant 5772708 of Kuopio University Hospital, and the Nordic Centre of Excellence in Neurodegeneration.

Italian sample collections : the Bologna site (FL) obtained funds from the Italian Ministry of research and University as well as Carimonte Foundation. The Florence site was supported by grant RF-2010-2319722 , grant from the the Cassa di Risparmio di Pistoia e Pescia (Grant 2012) and the Cassa di Risparmio di Firenze (Grant 2012). The Milan site was supported by a grant from the «fondazione Monzino» . We thank the expert contribution of Mr. Carmelo Romano. The Roma site received financial support from Italian Ministry of Health, Grant RF07-08 and RC08-09-10-11-12. The Pisa site is grateful to Dr Annalisa LoGerfo for her technical assistance in the DNA purification studies.

Spanish sample collection : the Madrid site (MB) was supported by grants of the Ministerio de Educación y Ciencia and the Ministerio de Sanidad y Consumo (Instituto de Salud Carlos III), and an institutional grant of the Fundación Ramón Areces to the CBMSO. We thank I. Sastre and Dr. A Martínez-García for the preparation and control of the DNA collection, and Drs. P. Gil and P. Coria for their cooperation in the cases/controls recruitment. We are grateful to the Asociación de Familiares de Alzheimer de Madrid (AFAL) for continuous encouragement and help.

Swedish sample collection : Financially supported in part by the Swedish Brain Power network, the Marianne and Marcus Wallenberg Foundation, the Swedish Research Council (521-2010-3134), the King Gustaf V and Queen Victoria’s Foundation of Freemasons, the Regional Agreement on Medical Training and Clinical Research (ALF) between Stockholm County Council and the Karolinska Institutet, the Swedish Brain Foundation and the Swedish Alzheimer Foundation.

**CHARGE**

AGES: The AGES-Reykjavik Study is funded by NIH contract N01-AG-12100 (NIA with contributions from the NEI, NIDCD and NHLBI), the NIA Intramural Research Program, Hjartavernd (the Icelandic Heart Association), and the Althingi (the Icelandic Parliament).

ASPS/PRODEM: The Austrian Stroke Prevention Study and The Prospective Dementia Register of the Austrian Alzheimer Society was supported by The Austrian Science Fond (FWF) grant number P20545-P05 (H. Schmidt) and P13180; The Austrian Alzheimer Society; The Medical University of Graz. Cardiovascular Health Study (CHS): This CHS research was supported by NHLBI contracts HHSN268201200036C, HHSN268200800007C, N01HC55222, N01HC85079, N01HC85080, N01HC85081, N01HC85082, N01HC85083, N01HC85086, and HHSN268200960009C; and NHLBI grants HL080295, HL087652, HL105756 with additional contribution from the National Institute of Neurological Disorders and Stroke (NINDS). Additional support was provided through AG023629, AG15928, AG20098, AG027058 and AG033193 (Seshadri) from the National Institute on Aging (NIA). A full list of CHS investigators and institutions can be found at http://www.chs-nhlbi.org/pi. The provision of genotyping data was supported in part by the National Center for Advancing Translational Sciences, CTSI grant UL1TR000124, and the National Institute of Diabetes and Digestive and Kidney Disease Diabetes Research Center (DRC) grant DK063491 to the Southern California Diabetes Endocrinology Research Center.

Framingham Heart Study (FHS): This work was supported by the National Heart, Lung and Blood Institute’s Framingham Heart Study (Contract No. N01-HC-25195) and its contract with A_ymetrix, Inc for genotyping services (Contract No. N02-HL-6-4278). A portion of this research utilized the Linux Cluster for Genetic Analysis (LinGA-II) funded by the Robert Dawson Evans Endowment of the Department of Medicine at Boston University School of Medicine and Boston Medical Center. This study as also supported by grants from the National Institute on Aging: AG08122 and AG033193 (Seshadri). Drs. Seshadri and DeStefano were also supported by additional grants from the National Institute on Aging: (R01 AG16495; AG031287, AG033040), the National Institute of Neurological Disorders and Stroke (R01 NS17950), and the National Heart, Lung and Blood Institute (U01 HL096917, HL093029 and K24HL038444, RC2-HL102419 and UC2 HL103010.

Fundació ACE would like to thank patients and controls who participated in this project. This work has been funded by the Fundación Alzheimur (Murcia), the Ministerio de Educación y Ciencia (PCT-010000-2007-18), (DEX-580000-2008-4), (Gobierno de España), Corporación Tecnológica de Andalucía (08/211) and Agencia IDEA (841318) (Consejería de Innovación, Junta de Andalucía). We thank to Ms. Trinitat Port-Carbó and her family for their generous support of Fundació ACE research programs.

The Rotterdam Study: The Rotterdam Study was funded by Erasmus Medical Center and Erasmus University, Rotterdam; the Netherlands Organization for Health Research and Development; the Research Institute for Diseases in the Elderly; the Ministry of Education, Culture and Science; the Ministry for Health, Welfare and Sports; the European Commission; and the Municipality of Rotterdam; by grants from the Research Institute for Diseases in the Elderly (014-93-015; RIDE2), Internationale Stichting Alzheimer Onderzoek, Hersenstichting Nederland, the Netherlands Genomics Initiative–Netherlands Organization for Scientific Research (Center for Medical Systems Biology and the Netherlands Consortium for Healthy Aging), the Seventh Framework Program (FP7/2007-2013), the ENGAGE project (grant agreement HEALTH-F4-2007-201413), MRACE-grant from the Erasmus Medical Center, the Netherlands Organization for Health Research and Development (ZonMW Veni-grant no. 916.13.054).

ARIC: The Atherosclerosis Risk in Communities Study (ARIC) is carried out as a collaborative study supported by National Heart, Lung, and Blood Institute contracts N01-HC-55015, N01-HC-55016, N01-HC-55018, N01- HC-55019, N01-HC-55020, N01-HC-55021, N01-HC-55022 and grants R01-HL087641, RC2-HL102419 (Boerwinkle, CHARGE-S), UC2 HL103010, U01-HL096917 (Mosley) and R01-HL093029; NHGRI contract U01- HG004402; and NIH contract HHSN268200625226C and NIA: R01 AG033193 (Seshadri). Infrastructure was partly supported by Grant Number UL1RR025005, a component of the National Institutes of Health and NIH Roadmap for Medical Research.

**GERAD**

Cardiff University was supported by theWellcome Trust, Medical Research Council (MRC), Alzheimer’s Research UK (ARUK) and the Welsh Government. ARUK supported sample collections at the Kings College London, the South West Dementia Bank, Universities of Cambridge, Nottingham, Manchester and Belfast. The Belfast group acknowledges support from the Alzheimer’s Society, Ulster Garden Villages, N.Ireland R&D Office and the Royal College of Physicians/Dunhill Medical Trust. The MRC and Mercer’s Institute for Research on Ageing supported the Trinity College group. DCR is a Wellcome Trust Principal Research fellow. The South West Dementia Brain Bank acknowledges support from Bristol Research into Alzheimer’s and Care of the Elderly. The Charles Wolfson Charitable Trust supported the OPTIMA group. Washington University was funded by NIH grants, Barnes Jewish Foundation and the Charles and Joanne Knight Alzheimer’s Research Initiative. Patient recruitment for the MRC Prion Unit/UCL Department of Neurodegenerative Disease collection was supported by the UCLH/UCL Biomedical Centre and their work was supported by the NIHR Queen Square Dementia BRU. LASER-AD was funded by Lundbeck SA. The Bonn group would like to thank Dr. Heike Koelsch for her scientific support. The Bonn group was funded by the German Federal Ministry of Education and Research (BMBF): Competence Network Dementia (CND) grant number 01GI0102, 01GI0711, 01GI0420. The AgeCoDe study group was supported by the German Federal Ministry for Education and Research grants 01 GI 0710, 01 GI 0712, 01 GI 0713, 01 GI 0714, 01 GI 0715, 01 GI 0716, 01 GI 0717. The Homburg group was funded by the German Federal Ministry of Education and Research (BMBF): German National Genome Research Network (NGFN); Alzheimer's disease Integrated Genome Research Network; AD-IG: 01GS0465. Genotyping of the Bonn case-control sample was funded by the German centre for Neurodegenerative Diseases (DZNE), Germany. The GERAD Consortium also used samples ascertained by the NIMH AD Genetics Initiative. HH was supported by a grant of the Katharina-Hardt-Foundation, Bad Homburg vor der Höhe, Germany. The KORA F4 studies were financed by Helmholtz Zentrum München; German Research Center for Environmental Health; BMBF; German National Genome Research Network and the Munich Center of Health Sciences. The Heinz Nixdorf Recall cohort was funded by the Heinz Nixdorf Foundation (Dr. Jur. G.Schmidt, Chairman) and BMBF. Coriell Cell Repositories is supported by NINDS and the Intramural Research Program of the National Institute on Aging. We acknowledge use of genotype data from the 1958 Birth Cohort collection, funded by the MRC and the Wellcome Trust which was genotyped by the Wellcome Trust Case Control Consortium and the Type-1 Diabetes Genetics Consortium, sponsored by the National Institute of Diabetes and Digestive and Kidney Diseases, National Institute of Allergy and Infectious Diseases, National Human Genome Research Institute, National Institute of Child Health and Human Development and Juvenile Diabetes Research Foundation International. The Nottingham Group (KM) are supported by the Big Lottery. MRC CFAS is part of the consortium and data will be included in future analyses.

**ADGC**

The National Institutes of Health, National Institute on Aging (NIH-NIA) supported this work through the following grants: ADGC, U01 AG032984, RC2 AG036528; NACC, U01 AG016976; NCRAD, U24 AG021886; NIA LOAD, U24 AG026395, R01 AG041797; MIRAGE R01 AG025259; Banner Sun Health Research Institute P30 AG019610; Boston University, P30 AG013846, U01 AG10483, R01 CA129769, R01 MH080295, R01 AG017173, R01AG33193; Columbia University, P50 AG008702, R37 AG015473; Duke University, P30 AG028377, AG05128; Emory University, AG025688; Group Health Research Institute, UO1 AG06781, UO1 HG004610; Indiana University, P30 AG10133; Johns Hopkins University, P50 AG005146, R01 AG020688; Massachusetts General Hospital, P50 AG005134; Mayo Clinic, P50 AG016574; Mount Sinai School of Medicine, P50 AG005138, P01 AG002219; New York University, P30 AG08051, MO1RR00096, and UL1 RR029893; Northwestern University, P30 AG013854; Oregon Health & Science University, P30 AG008017, R01 AG026916; Rush University, P30 AG010161, R01 AG019085, R01 AG15819, R01 AG17917, R01 AG30146; TGen, R01 NS059873; University of Alabama at Birmingham, P50 AG016582, UL1RR02777; University of Arizona, R01 AG031581; University of California, Davis, P30 AG010129; University of California, Irvine, P50 AG016573, P50, P50 AG016575, P50 AG016576, P50 AG016577; University of California, Los Angeles, P50 AG016570; University of California, San Diego, P50 AG005131; University of California, San Francisco, P50 AG023501, P01 AG019724; University of Kentucky, P30 AG028383; University of Michigan, P50 AG008671; University of Pennsylvania, P30 AG010124; University of Pittsburgh, P50 AG005133, AG030653, AG041718; University of Southern California, P50 AG005142; University of Texas Southwestern, P30 AG012300; University of Miami, R01 AG027944, AG010491, AG027944, AG021547, AG019757; University ofWashington, P50 AG005136; Vanderbilt University, R01 AG019085; andWashington University, P50 AG005681, P01 AG03991. The Kathleen Price Bryan Brain Bank at Duke University Medical Center is funded by NINDS grant # NS39764, NIMH MH60451 and by Glaxo Smith Kline. Genotyping of the TGEN2 cohort was supported by Kronos Science. The TGen series was also funded by NIA grant AG034504 to AJM, The Banner Alzheimer’s Foundation, The Johnnie B. Byrd Sr. Alzheimer’s Institute, the Medical Research Council, and the state of Arizona and also includes samples from the following sites: Newcastle Brain Tissue Resource (funding via the Medical Research Council, local NHS trusts and Newcastle University), MRC London Brain Bank for Neurodegenerative Diseases (funding via the Medical Research Council), South West Dementia Brain Bank (funding via numerous sources including the Higher Education Funding Council for England (HEFCE), Alzheimer’s Research Trust (ART), BRACE as well as North Bristol NHS Trust Research and Innovation Department and DeNDRoN), The Netherlands Brain Bank (funding via numerous sources including Stichting MS Research, Brain Net Europe, Hersenstichting Nederland Breinbrekend Werk, International Parkinson Fonds, Internationale Stiching Alzheimer Onderzoek), Institut de Neuropatologia, Servei Anatomia Patologica, Universitat de Barcelona. Marcelle Morrison-Bogorad, PhD., Tony Phelps, PhD and Walter Kukull PhD are thanked for helping to co-ordinate this collection. ADNI Funding for ADNI is through the Northern California Institute for Research and Education by grants from Abbott, AstraZeneca AB, Bayer Schering Pharma AG, Bristol-Myers Squibb, Eisai Global Clinical Development, Elan Corporation, Genentech, GE Healthcare, Glaxo-SmithKline, Innogenetics, Johnson and Johnson, Eli Lilly and Co., Medpace, Inc., Merck and Co., Inc., Novartis AG, Pfizer Inc, F. Hoffman-La Roche, Schering-Plough, Synarc, Inc., Alzheimer’s Association, Alzheimer’s Drug Discovery Foundation, the Dana Foundation, and by the National Institute of Biomedical Imaging and Bioengineering and NIA grants U01 AG024904, RC2 AG036535, K01 AG030514. Data collection and sharing for this project was funded by the ADNI (National Institutes of Health Grant U01 AG024904). ADNI is funded by the National Institute on Aging, the National Institute of Biomedical Imaging and Bioengineering, and through generous contributions from the following: Alzheimer’s Association; Alzheimer’s Drug Discovery Foundation; BioClinica, Inc.; Biogen Idec Inc.; Bristol-Myers Squibb Company; Eisai Inc.; Elan Pharmaceuticals, Inc.; Eli Lilly and Company; F. Hoffmann-La Roche Ltd and its affiliated company Genentech, Inc.; GE Healthcare; Innogenetics, N.V.; IXICO Ltd.; Janssen Alzheimer Immunotherapy Research & Development, LLC.; Johnson & Johnson Pharmaceutical Research & Development LLC.; Medpace, Inc.; Merck & Co., Inc.; Meso Scale Diagnostics, LLC.; NeuroRx Research; Novartis Pharmaceuticals Corporation; Pfizer Inc.; Piramal Imaging; Servier; Synarc Inc.; and Takeda Pharmaceutical Company. The Canadian Institutes of Health Research is providing funds to support ADNI clinical sites in Canada. Private sector contributions are facilitated by the Foundation for the National Institutes of Health (www.fnih.org). The grantee organization is the Northern California Institute for Research and Education, and the study is coordinated by the Alzheimer’s Disease Cooperative Study at the University of California, San Diego. ADNI data are disseminated by the Laboratory for Neuro Imaging at the University of California, Los Angeles. This research was also supported by NIH grants P30 AG010129 and K01 AG030514. We thank Drs. D. Stephen Snyder and Marilyn Miller from NIA who are ex-o_cio ADGC members. Support was also from the Alzheimer’s Association (LAF, IIRG-08-89720; MP-V, IIRG-05-14147) and the US Department of Veterans Affairs Administration, Office of Research and Development, Biomedical Laboratory Research Program. P.S.G.-H. is supported by Wellcome Trust, Howard Hughes Medical Institute, and the Canadian Institute of Health.
